# Scientometric analysis of extracellular vesicles in vision science (up to 2024)

**DOI:** 10.1186/s12951-025-03703-5

**Published:** 2025-10-10

**Authors:** Fei Liao, Francisco Germain, Li Ma, Chao Wei, Ting Wang

**Affiliations:** 1 Eye Hospital of Shandong First Medical University (Shandong Eye Hospital), Eye Institute of Shandong First Medical University, State Key Laboratory Cultivation Base, Shandong Key Laboratory of Eye Diseases, School of Ophthalmology, Shandong First Medical University, Jinan, 250021 China; 2 State Key Laboratory Cultivation Base, Shandong Key Laboratory of Eye Diseases, Eye Institute of Shandong First Medical University, School of Ophthalmology, Qingdao Eye Hospital of Shandong First Medical University, Qingdao , 266071 China; 3 Department of Systems Biology, Laboratory of Visual Neurophysiology, University of Alcalá, 28871 Alcalá de HenaresMadrid, Spain; 4 Instituto Ramón y Cajal de Investigación Sanitaria (IRYCIS), Hospital Ramón y Cajal, 28034 Madrid, Spain

**Keywords:** Extracellular vesicles, Vision science, Biomarker, MSCs, Visualization, Cornea, Retina, RPE, Glaucoma

## Abstract

**Background:**

Extracellular vesicles (EVs) in vision science have gained significant attention. However, a comprehensive scientometric analysis of the key major contributors, the current research landscape, and development trends is still lacking.

**Objectives:**

To detect and visualize the research strengths, knowledge base, and research frontiers of EVs in vision science.

**Methods:**

Publications of EVs on vision science were systematically collected from Web of Science, PubMed, Scopus, and Embase, covering the inception of each database up to December 31, 2024. Following data cleaning, a bibliometric assessment was conducted primarily based on VOSviewer and CiteSpace platforms. Key analysis included temporal publication trends, co-authorship patterns, the knowledge base, and the thematic evolution of research trends.

**Results:**

A total of 427 original research articles were analyzed, with an average of 25.69 citations per article and an H-index of 55. The global annual cumulative publication showed exponential growth across three phases: a silent period (2003–2012, 8 articles), gradual growth (2013–2019, 67 articles), and a sharp surge from 2020 onward (352 articles). United States and China led in publication output, with the University of California System emerging as the most collaborative institution. The knowledge base comprises thirteen well-established themes, which originated around 2009. Fifteen salient research frontier themes have emerged, most of which remain in the developmental phase.

**Conclusion:**

The roles of EVs in pathophysiology, diagnostic, and therapeutic potential in vision science have been extensively explored; however, notable limitations and gaps remain, warranting further investigation. Moreover, the clinical translation of EVs-based applications faces significant challenges.

**Graphical abstract:**

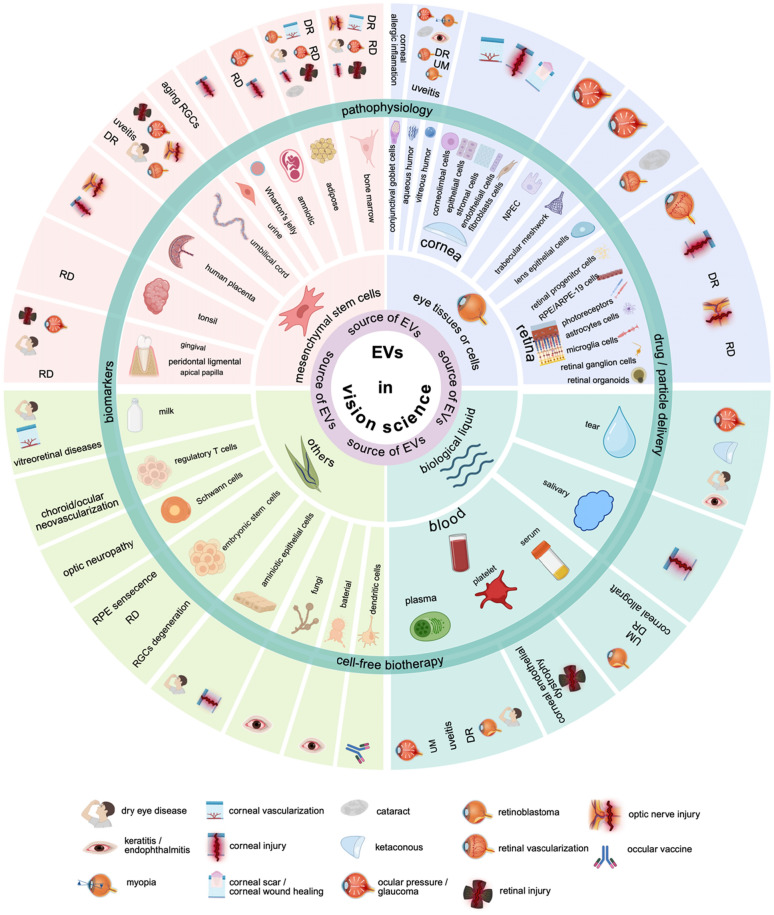

**Supplementary Information:**

The online version contains supplementary material available at 10.1186/s12951-025-03703-5.

## Introduction

In recent years, extracellular vesicles (EVs) have gained increasing attention in vision science due to their promising diagnostic and therapeutic potentials. These nano-sized, bilayer lipid-bound vesicles are naturally secreted by cells into the extracellular space under physiological or pathological states. Based on their biogenesis, release pathways, size, content, and function, EVs consist of three main subtypes, including microvesicles (100 nm – 1 μm), exosomes (30–150 nm), and apoptotic bodies (50–5000 nm) [1, 2].

EVs contain a diversity of cargo, such as proteins, lipids, nucleic acids, and/or metabolites. These enable them to communicate among cells and regulate biological processes, such as the immune response, tissue repair, angiogenesis, etc. [3]. Thereby, EVs provide new insights into the pathophysiology and biomarker discovery [4]. Moreover, EVs exhibit good biocompatibility, stability, and a high capacity to traverse biological barriers, making them promising candidates for drug delivery and innovative treatment strategies [4, 5].

While publications on EVs in vision science are proliferating rapidly, no cohesive bibliometric assessment has evaluated their evolution trajectory. To date, one study systematically evaluated this area [6]; however, it relied solely on documents from the Web of Science (WoS), with a period from 2003 to 2022, and even inapropriately included reviews, which could result in discrepancies.

Bibliometrics is a widely recognized method that facilitates the analysis of scientific progress on a given topic [7, 8]. Accordingly, in this work, we apply the scientometric technique to assess the effectiveness and productivity of research efforts, the knowledge base, the research frontiers, and potential research gaps in the field of EVs in vision science. By analyzing the primary scientific output of original research articles collected from four globally recognized databases (WoS, Scopus, Embase, and PubMed) covering publications up to 31 December 2024, this work has the potential to serve as a contemporary resource to enhance readers´ comprehension of this field and to provide directions for pursuing collaboration or addressing research gaps.

## Materials and methods

### Search strategy of literature source

Following the identification of MeSH Heading subjects by PubMed, on January 14, 2025, a systematic literature source was collected online through WoS (Core Collection, Science Citation Index Expanded), Scopus, Embase, and PubMed, which are four globally recognized databases. The search aimed to identify publications related to EVs in vision science. The following base Boolean search strings format was used, adapted as needed for each databases syntax and field codes: (exosome* OR microvesicle* OR "extracellular vesicle*") AND (eye OR eyebrow* OR eyelid* OR eyelash* OR "anterior eye segment" OR "anterior chamber" OR "aqueous humor" OR "ciliary body" OR conjunctiva OR cornea OR iris OR lens OR "meibomian glands" OR "lacrimal apparatus" OR "nasolacrimal duct" OR "pigment epithelium" OR "posterior eye segment" OR "vitreous humor" OR retina OR "blood-retinal barrier" OR "macula lutea" OR "optic disk" OR "retinal neurons" OR sclera OR "tenon capsule" OR uvea OR "blood-aqueous barrier" OR choroid OR "optic nerve"). Searching was confined to the title, abstract, and author keywords, with the publication date range from each databases inception by December 31, 2024. No additional filters were applied. Full search strings for each database are available in the supplementary methods section.

### Data integration, inclusion, and exclusion criteria

The metadata from four databases were first integrated using Microsoft Excel 2021. Duplicate records identified by title and authors were removed before screening. Non-article publication types were removed, including review, meta-analysis, editorial, letter, book or book chapter, conference abstract, retraction, errata, protocol, preprint (e.g., bioRxiv), or prospective. Then, two authors independently reviewed all studies by title, abstract, and/or full text to remove irrelevant articles. Records focusing simultaneously on EVs and eyes were included, regardless of the source of EVs, whether derived from human models, non-human models, or organoids. Otherwise, records were excluded. Any disagreements were decided by a third author. For the included records retrieved from PubMed and Embase, items of author keywords, abstracts, affiliations, and funding agencies were manually completed, and citation counts were obtained from Google Scholar. This is because these items were absent in the exported dataset from these two databases.

### Data processing and primary analysis

Names of countries, authors, institutions, and journal sources were standardized by merging variations while unifying synonyms for author keywords (**Suppl. Table S1-S5**). Records were converted into the WoS tab-delimited format for knowledge mapping using four complementary platforms: VOSviewer (version 1.6.20), Pajek (version 5.18), CiteSpace (version 6.2.6 Advanced), and Bibliometrix R-package. Specifically, VOSviewer analyzed collaboration networks among countries, researchers, and institutions. Pajek refined and optimized node visualization from VOSviewer. CiteSpace examined the scientific distribution and its dynamic trajectories through a dual-journal map, mapped the knowledge base via cited reference analysis, and underscored the research frontiers via the author keyword co-occurrence. The Bibliometrix R-package was employed to identify core journal sources (specifically its Biblioshiny interface), and the temporal evolution of the top 30 most frequently occurring author keywords. Thematic cluster size is indicated by a numerical label, with smaller numbers corresponding to larger clusters.

Furthermore, CiteSpace was applied to detect the strongest citation burst and the nodes with high betweenness centrality (≥ 0.1, marked in purple). The former indicates research hotspots and/or emerging trends. The latter are generally referred to as critical hubs or "turning points" whose removal would significantly disrupt network integrity and transmission efficiency. In certain cases, the sigma value, an index that integrates both the frequency of occurrence and betweenness centrality, was also detected using CiteSpace [8, 9].

CiteSpace settings are presented below: years per slice = 1, cosine strength, scope within slices, pathfinder pruning, and g-index scale factors of k = 50 for cited references and k = 100 for author keywords. After filtering, 365 author keywords were finally obtained for analysis. To assess clustering quality, the Modularity (Q) and Silhouette (S) values, ranging from 0 to 1, are commonly used. A Q value > 0.3 indicates a significant community structure (> 0.5 are generally acceptable), while an S value closer to 1 reflects higher homogeneity [9, 10]. In this work, the clustering results demonstrated strong community structure and high homogeneity, with Q = 0.9233 and S = 0.9677 for the cluster of the knowledge base from cited references, and Q = 0.8692 and S = 0.9692 for the cluster of research frontiers from author keywords.

## Results

**Fig. 1:**
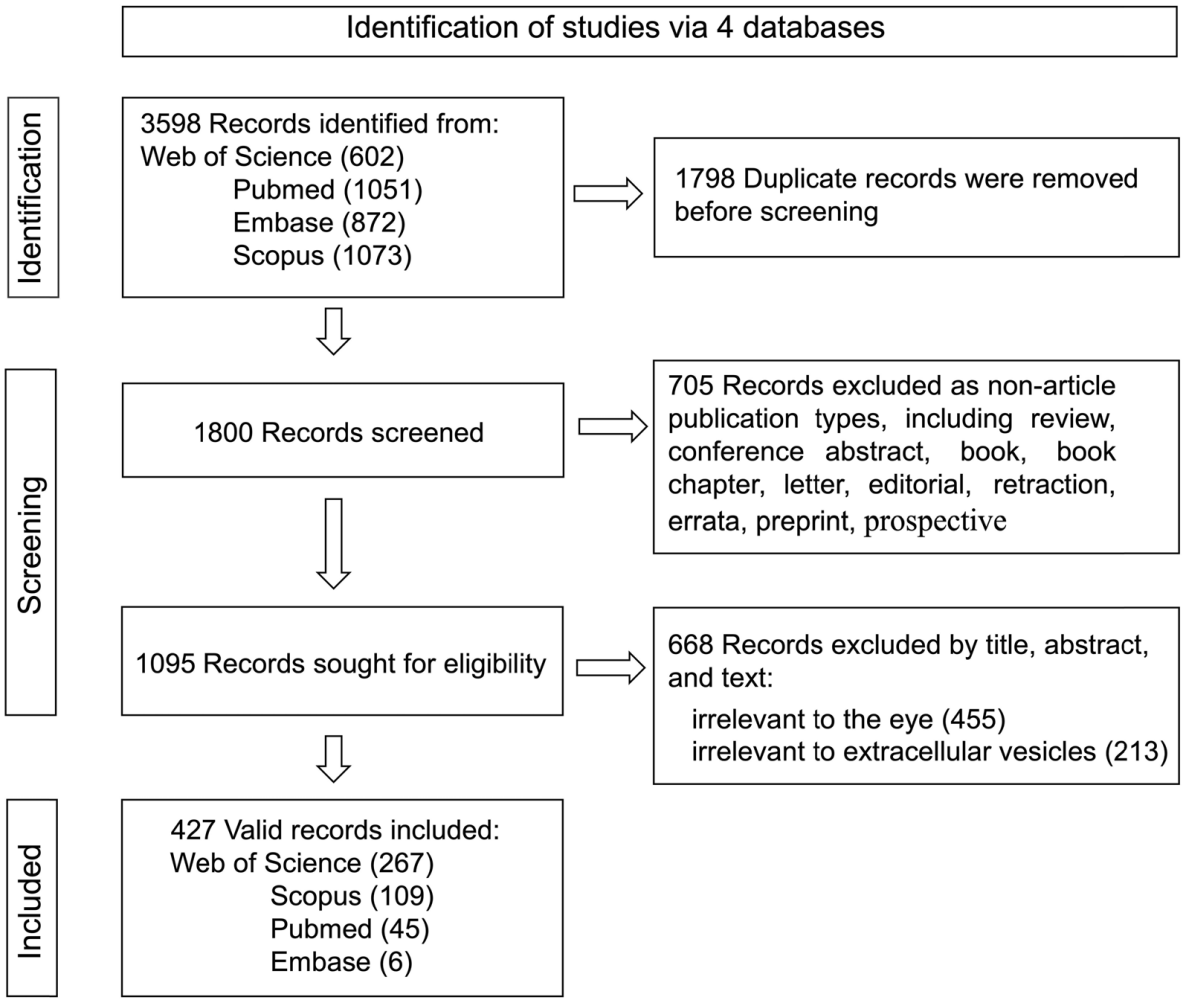
Flowgram of data collection and cleanning for included studies

A total of 3,598 literature records were identified through four databases. After removing 1,798 duplicate records, 427 valid documents were finally retained following data cleaning (Fig. 1), covering the timespan from 2003 to 2024. Among them, WoS yielded 267 publications, Scopus 109, PubMed 45, and Embase 6. The dataset encompasses 2,549 authors affiliated with 540 organizations across 38 countries. It includes 17,644 cited references published in 2,913 journals, collectively receiving 21,953 referenced times. Moreover, the included 427 original research articles have received a cumulative number of 10,972 citations, with an average of 25.69 citations per article, an H-index of 55, and the top 50 papers contribute 48.73% of total citations.

### Trend in publications

**Fig. 2:**
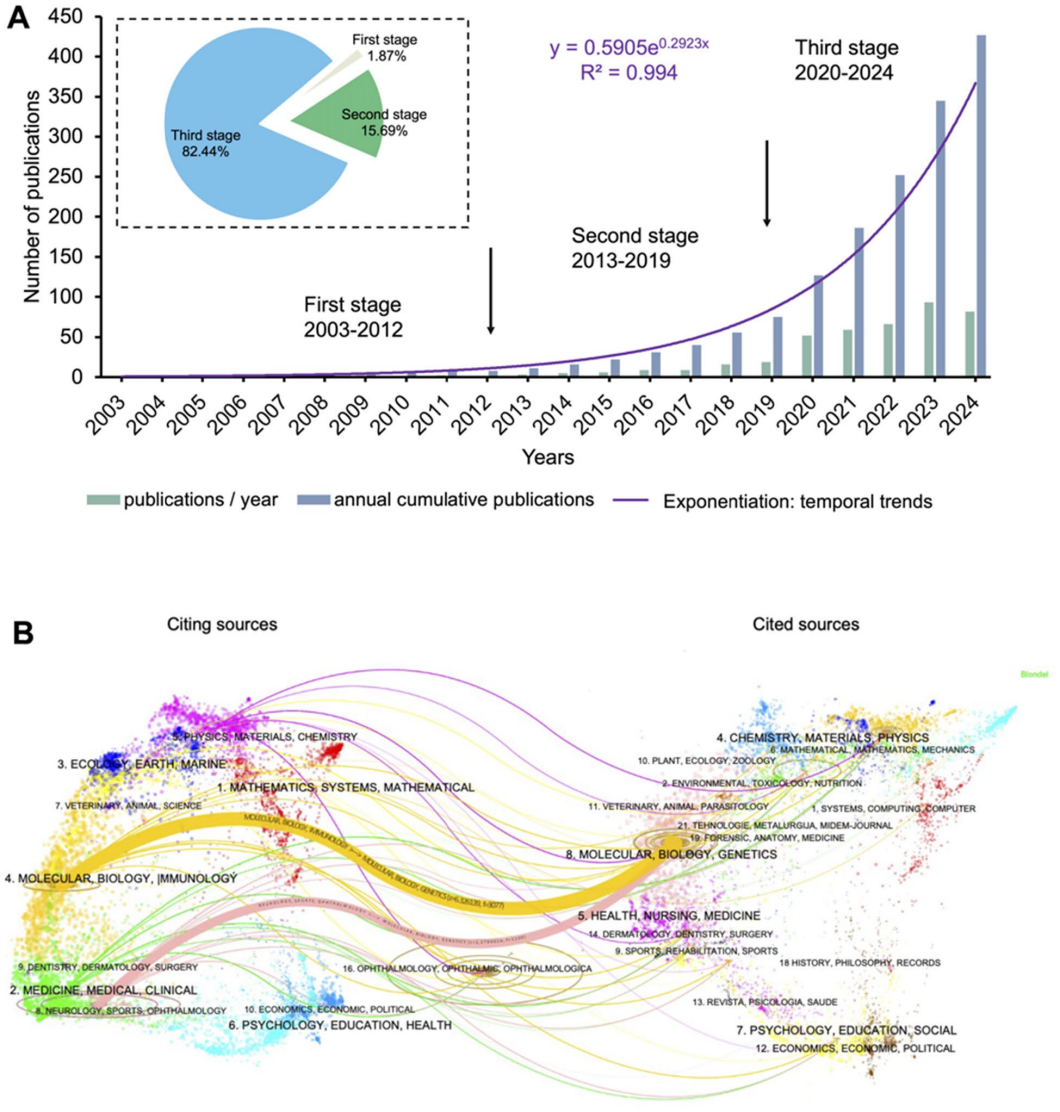
**(A)** Temporal distribution of publications from 2003 to 2024. **(B)** Journal dual-map overlay

We sorted the number of articles by year (Fig. 2A). Of note, although we collected publication dates starting from the inception of each database, the earliest relevant study was yielded in 2003 [11]. This indicated the beginning of EVs research in vision science. Subsequently, the second and third relevant articles were yielded in 2005 [12] and 2007 [13], respectively, both focusing on myocilin-associated EVs research.

The annual publication trend follows three stages. The first stage: a silent period (2003–2012, 8 papers), annual publications ≤ 2, with occasional years (2004, 2006, 2008, 2012) lacking new contributions. The second stage (2013–2019, 67 papers) shows gradual growth but stays below 20 annually. The third stage: a sharp surge from 2020 onward (352 papers), publications surpassed 50 articles/year. Overall, the accumulative global publication follows an exponential growth curve f(x) = [0.5905*e(0.2923*x), R^2^ = 0.994], poising that sustained growth in the coming years.

### Distribution of subject categories

As illustrated by the journal dual-map overlay (Fig. 2B), the cited sources indicate that this fields knowledge base is primarily rooted in molecular biology and genetics. Over time, however, the citing sources reveal a shift in this knowledge base, with trajectories extending toward the clusters of molecular biology and immunology, as well as neurology and ophthalmology.

An in-depth analysis of the included articles shows that they encompassed 50 subject categories, which are led by ophthalmology (96 papers, 22.48%), cell biology (88 papers, 20.61%), and biochemistry & molecular biology (53 papers, 12.41%). Notably, biochemistry & molecular biology also demonstrated the highest sigma value (1.66), indicating its critical role in the foundation and hub for advancing EVs research in ophthalmology.

### Coauthorship network

#### Global cooperation and focus themes

**Fig. 3:**
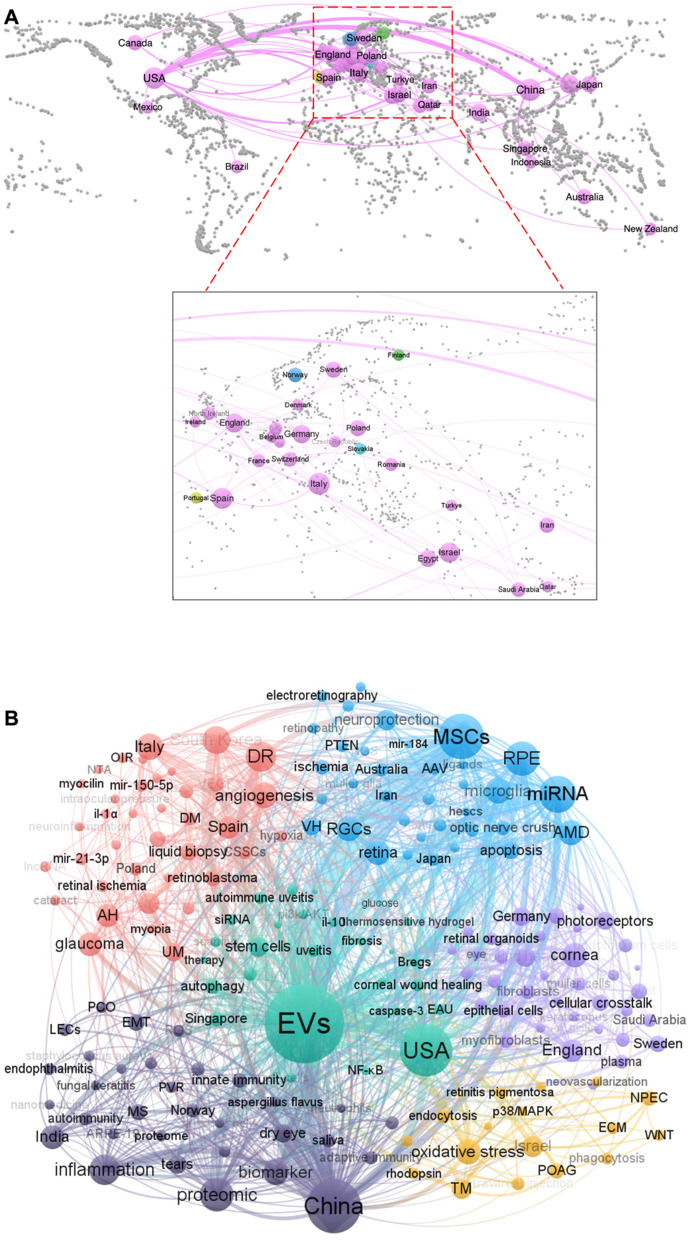
Coauthorship network among countries: **(A)** Global collaboration, **(B)** Countries´ focus theme

United States and China are the most prolific countries and have demonstrated the most vigorous global collaboration (Fig. 3A). China partnered with 21 countries and has produced the most publications, with 185 articles Table 1. The United States collaborated with 21 countries and strongly partnered with England, Germany, Spain, Sweden, and South Korea. Furthermore, it holds a leading position in total and average citations. Notably, 13 countries (34.21%) collaborated with only one partner, such as Japan, Brazil, Qatar, Denmark, Iran, and Mexico. These findings underscore the need to strengthen international research cooperation.

**Table 1:** The top 10 productive countries

Country	N	Total Citations	Average Citations	%	H-index
China	182	3678	20.21	42.62	38
USA	135	4656	34.49	31.62	38
South Korea	32	756	23.63	7.49	13
Italy	21	676	32.19	4.92	15
India	18	92	5.11	4.22	7
Spain	17	623	36.65	3.98	12
Israel	13	271	20.85	3.04	9
England	11	203	18.45	2.58	8
Germany	9	131	14.56	2.11	6
Japan	8	163	20.38	1.87	8

Research focus varies by country (Fig. 3B). Particularly, the United States and Singapore prioritize stem cells, uveitis, corneal wound healing, autophagy, and epithelium (green cluster). China, India, and Norway focus on proteomics, inflammation, and biomarkers (dark purple cluster). Italy, Poland, Spain, and South Korea investigate diabetic retinopathy, glaucoma, aqueous humor, angiogenesis, and uveal melanoma (pink cluster). Iran and Japan study mesenchymal stem cells (MSCs), retinal pigment epithelium (RPE), microRNA (miRNA), age-related macular degeneration (AMD), microglia, and retina (blue cluster). England, Germany, and Sweden emphasize wound healing, cornea, and photoreceptors (light purple cluster), while Israel researches oxidative stress, trabecular meshwork (TM), and non-pigment epithelial cell (NPEC, yellow cluster).

### Core authorship network and focus theme

The authorship of the literature was analyzed to identify key scholars and core research forces. The most prolific author published a maximum of n_max_ = 18 articles. According to Prices Law, a core author is defined as one who has published at least m = 0.749x√n_max_≈3. Based on this criterion, 173 core authors were identified, collectively contributing 240 papers, which account for more than half, concrete, 56.21%, of the total papers. Herein, a relatively stable group of researchers has been established in this field.

**Fig. 4:**
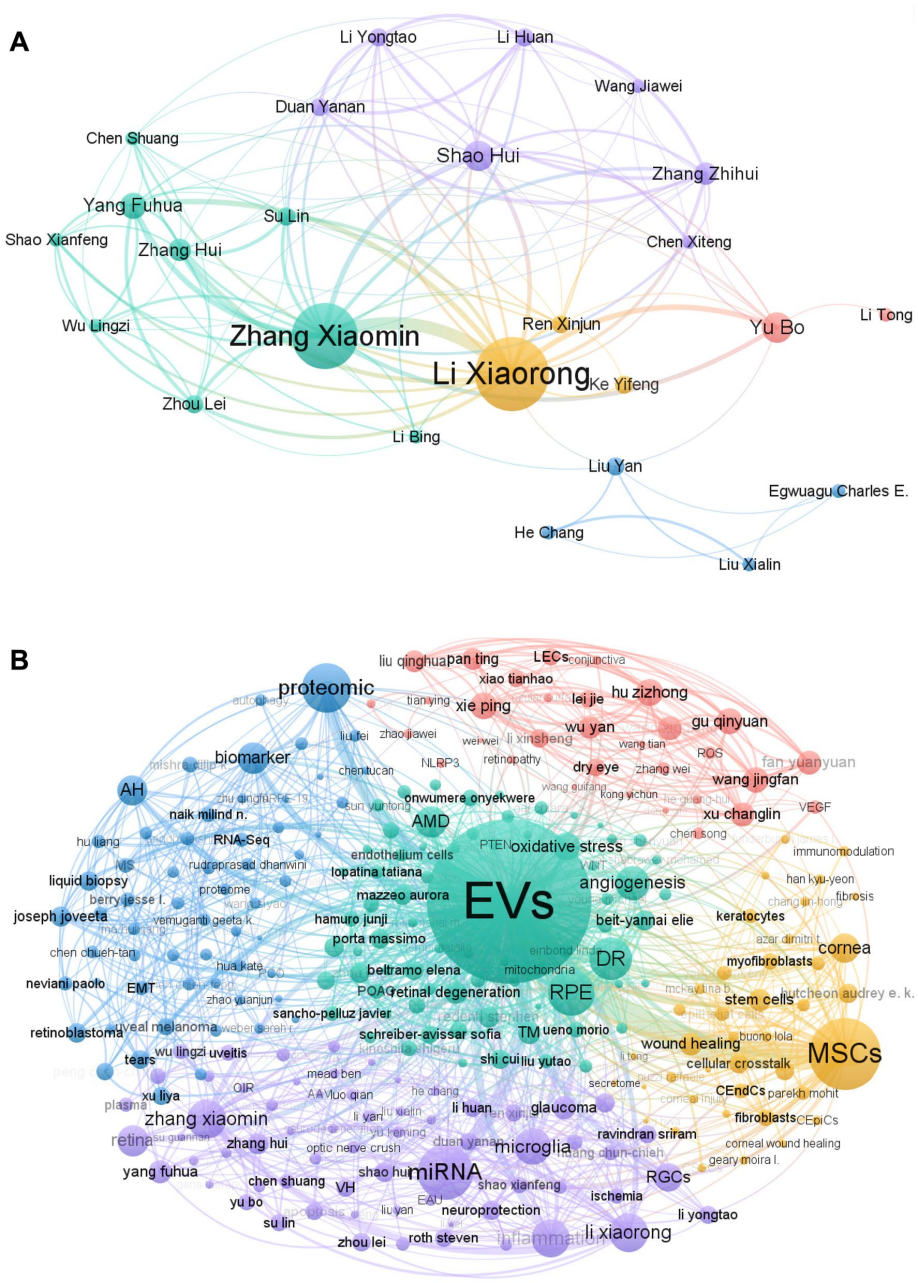
Coauthorship network among core authors: **(A)** Core authors collaboration, **(B)** Core authors´ focus theme

Nonetheless, only 32 core authors have established collaborative networks, comprising five clusters (Fig. 4A). The central figures of clusters 1, 4, and 5 correspond with Zhang Xiaomin, Li Xiaorong, and Yu Bo, respectively, are affiliated with Tianjin Medical University (China). Shao Hui is the central figure of Cluster 2 and is associated with the University of Louisville (USA). Cluster 3 is centered around Liu Yan from Shanghai Jiao Tong University (China). Notably, all these five central figures primarily focus on miRNA, microglia, glaucoma, and retina (Fig. 4B, light purple cluster).

### High‐contributing institutions and focus theme

Among the top 10 contributing institutions (Table 2), five are based in China, four in the United States, and one in India. Cumulatively, they have published 176 documents (41.22% of total publications) and accumulated 4,434 citations (25.13% of total citations).

**Table 2:** Top 10 institutions of high contribution

Institution	N	Total Citations	Average Citations	%	Earlist publication	H-index
Tianjin Med Univ (China)	31	1151	37.13	7.26	2016	17
Univ Of California System (USA)	23	581	25.26	5.39	2011	12
Harvard Med School (USA)	19	473	24.89	4.45	2017	12
Harvard Univ (USA)	18	473	26.28	4.22	2017	12
Sun Yat Sen Univ (China)	18	362	20.11	4.22	2021	9
Wenzhou Med Univ (China)	17	340	20.00	3.98	2018	8
L. V. Prasad Eye Institute (India)	14	44	3.14	3.28	2022	4
Capital Med Univ (China)	14	267	19.07	3.28	2020	8
Chinese Acad Sci (China)	11	358	32.55	2.58	2016	13
Duke Univ (USA)	11	385	35.00	2.58	2005	8

**Fig. 5:**
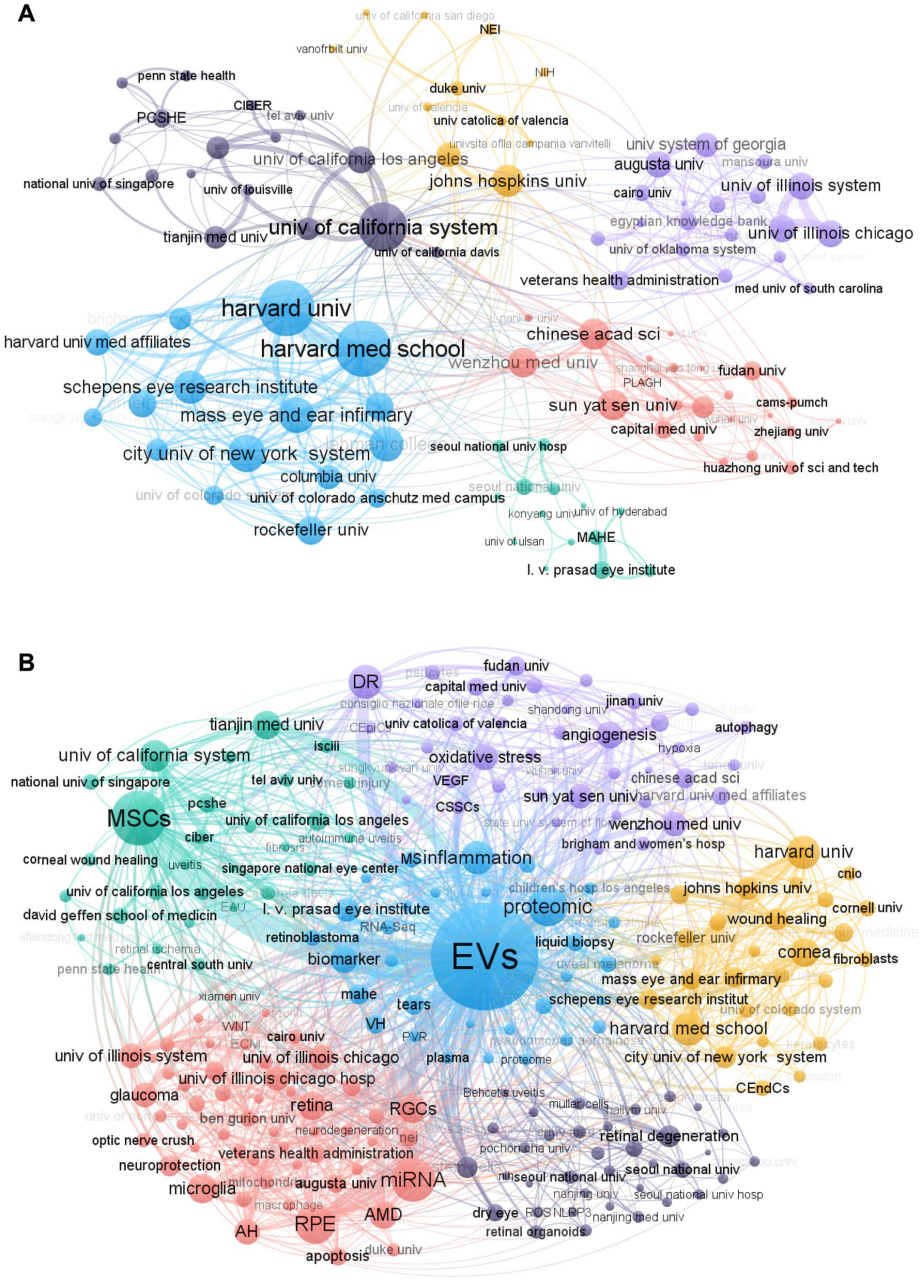
Coauthorship network among institutions: **(A)** Institutions collaboration, **(B)** Institutions´ focus theme

Tianjin Medical University (China) ranks first in publication volume (31 papers), total citations (1,151 citations), average citations per paper (37.13), and H-index [17]. Along with the University of California System, the most collaborative institution (Fig. 5A), their research primarily focuses on MSCs, corneal injury, and wound healing (Fig. 5B, green cluster). Duke University was the earliest contributor to this field, with its first publication in 2005, focusing on miRNA, RPE, AMD, and retina (Fig. 5B**,** pink cluster).

Harvard Medical School and Harvard University demonstrate the second and third highest levels of institutional collaboration, primarily researching the cornea and wound healing (Fig. 5B, yellow cluster). The Chinese Academy of Sciences ranks fourth in institutional collaboration, together with Capital Medical University, Sun Yat-sen University, and Wenzhou Medical University, with a primary focus on diabetic retinopathy, oxidative stress, and angiogenesis (Fig. 5B**,** light purple cluster). Moreover, the L.V. Prasad Eye Institute focuses on inflammation, proteomics, and biomarkers (Fig. 5B, blue cluster).

### High‐contributing funding agencies

The National Natural Science Foundation of China (NSFC, China) ranks first for supporting publications in this field, having Funded 106 studies, accounting for 24.82% of the total (Table 3). Subsequently, it was followed by the National Institutes of Health (NIH, USA) and the National Eye Institute (NEI, USA), supporting 77 (18.03%) and 43 (10.07%) studies, respectively. Among the top-ranking funding agencies, four are based in China, three in the United States, one in South Korea, one in Japan, and one in Israel.

**Table 3:** Top 10 funding agencies

Funding agency	N	%
National Natural Science Foundation of China (NSFC, China)	106	24.82
National Institutes of Health (NIH, USA)	77	18.03
National Eye Institute (NEI, USA)	43	10.07
Research to Prevent Blindness (RPB, USA)	32	7.49
National Research Foundation of Korea (South Korea, NRF)	13	3.04
National Key Research and Development Program of China (NKRDPC, China)	10	2.34
Hyderabad Eye Research Foundation (HERF, Japan)	10	2.34
Natural Science Foundation of Zhejiang Province (China)	7	1.64
Natural Science Foundation of Jiangsu Province (China)	8	1.87
Israel Science Foundation (Israel)	8	1.87

### High‐contributing and contributed journals

The top 10 citing journals (Table 4) are the same as those classified into Zone 1 based on Bradfords Law using the software Biblioshiny, indicating that they represent the core journal sources in the field. Collectively, they published 157 papers (36.77% of the total), highlighting the relatively high centralization of research outputs in a small group of journals, consistent with a developing yet increasingly focused research domain. The journal *Experimental Eye Research (EER)* had the most publications (34 papers), while *Investigative Ophthalmology & Visual Science (IOVS)* demonstrated the highest total citations (998 times) and average citations per paper (35.29). Both share an H-index of 15, reflecting a sustained academic impact over time. The top 10 cited journals collectively contributed 2,456 papers, with *IOVS* leading in publication volume (811 papers), total citations (1,094 times), average citations per paper (1.35), and an H-index of 8. *EER* followed with 422 papers and 556 citations.

**Table 4:** Top 10 citing and cited sources

	**Journal**	**N**	**%**	**Total citations**	**Average** **citations**	**H-index**	**JCR**	**Discipline**
**Citing sources**	EXP EYE RES	34	7.96	593	17.44	15	Q1	Ophthalmology
	INVEST OPHTH VIS SCI	28	6.56	988	35.29	15	Q1	Ophthalmology
	INT J MOL SCI	21	4.92	273	13.00	12	Q1	Biochem. & Mol. Biol
	CELLS-BASEL	15	3.51	165	11.00	7	Q2	Cell Biology
	SCI REP-UK	16	3.75	904	56.50	13	Q1	Multi. Sciences
	STEM CELL RES THER	9	2.11	249	27.67	8	Q1	Cell & Tis Engi
	EXP CELL RES	8	1.87	206	25.75	6	Q3	Cell Biology
	J CELL MOL MED	7	1.64	450	64.29	6	Q2	Cell Biology
	J EXTRACELL VESICLES	7	1.64	166	23.71	5	Q1	Cell Biology
	PLOS ONE	6	1.41	285	47.50	6	Q1	Multi. Sciences
**Cited sources**	INVEST OPHTH VIS SCI	811	4.60	1094	1.35	8	Q1	Ophthalmology
	EXP EYE RES	422	2.39	556	1.32	6	Q1	Ophthalmology
	PLOS ONE	358	2.03	446	1.25	5	Q1	Multi. Sciences
	SCI REP-UK	309	1.75	462	1.50	7	Q1	Multi. Sciences
	INT J MOL SCI	307	1.74	376	1.22	5	Q1	Biochem. & Mol. Biol
	P NATL ACAD SCI USA	266	1.51	332	1.25	3	Q1	Multi. Sciences
	Prog Retin Eye Res	241	1.37	410	1.70	6	Q1	Ophthalmology
	J BIOL CHEM	210	1.19	275	1.31	5	Q2	Biochem. & Mol. Biol
	J EXTRACELL VESICLES	172	0.97	349	2.03	7	Q1	Cell Biology
	STEM CELL RES THER	161	0.91	236	1.47	5	Q1	Cell & Tis. Engi

Biochem. & Mol. Biol., Biochemistry & Molecular Biology. Multi., Multidisciplinary. Cell & Tis. Engi., Cell & Tissue Engineering.

Notably, *EER* and *IOVS* are both vision science-oriented journals, indicating a strong interest in EVs within basic and translational in vision science. Meanwhile, the EVs-specialized journal, *Journal of Extracellular Vesicles*, ranked ninth either for high‐contributing or high-contributed journals. Of note, while representing only 0.97% of the total cited references, the *Journal of Extracellular Vesicles* recorded the highest average citations per paper (2.03). Overall, this trend indicates the fields growing cross-disciplinary integration, with ophthalmic EVs studies gaining recognition within core EVs research.

### Knowledge base

**Fig. 6:**
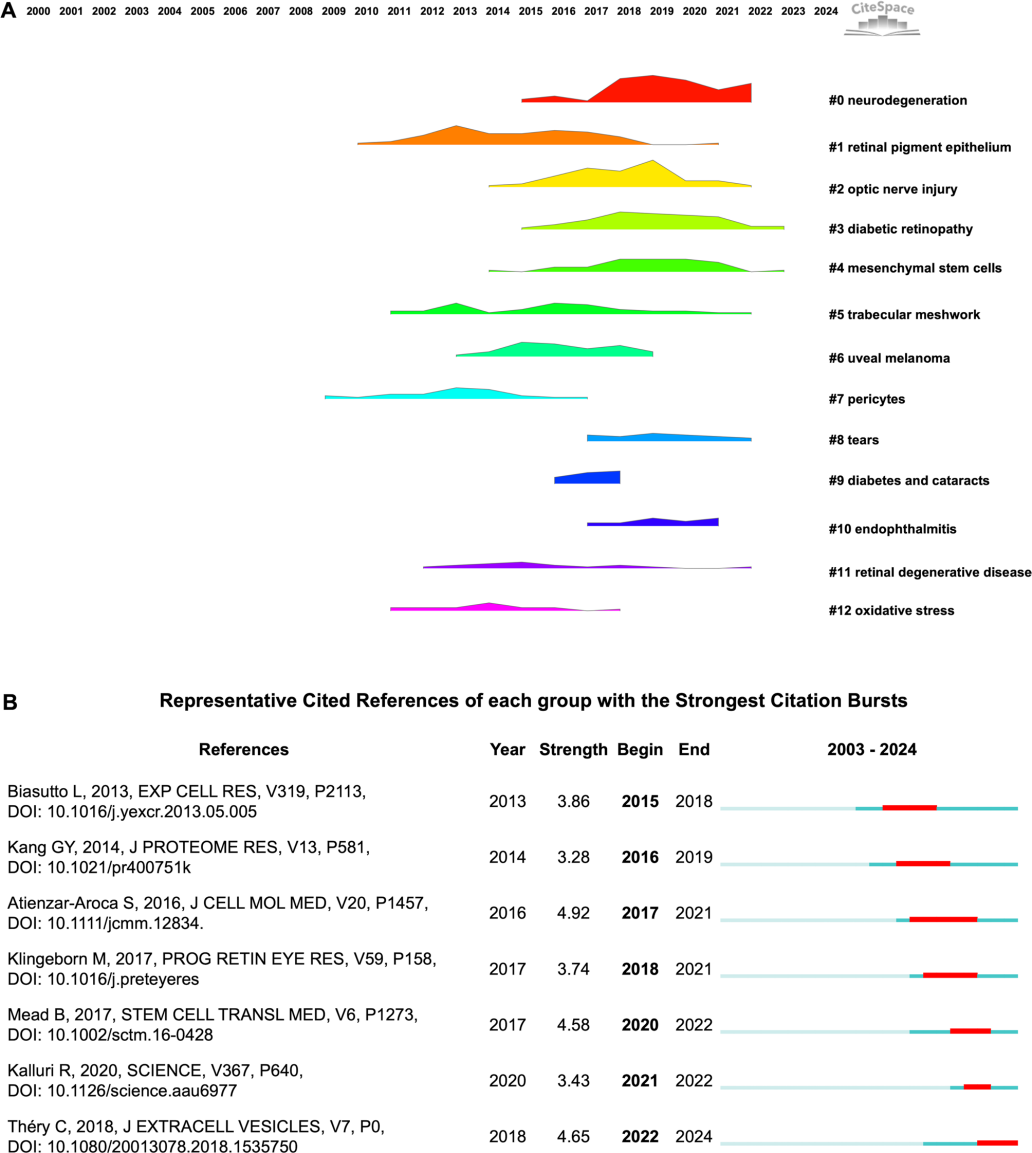
**(A)** Themetic landscape of the knowledge base. **(B)** The most representative cited reference from each annual cohort with the strongest citation bursts

To a given topic, the knowledge base, also referred to as the knowledge foundation, is fundamentally established through a collection of co-cited references. Using CiteSpace, we detected 13 thematic clusters representing the intellectual evolution of EVs research in vision science over time (Fig. 6A). While the earliest cited reference dates to 2000, the emergence of clusters began in 2009, labeled"pericytes". The second cluster, termed "RPE", began forming in 2010. Clusters "TM" and "oxidative stress" appeared in 2011, followed by "retinal degenerative disease" in 2012, "uveal melanoma" in 2013, "optic nerve injury" and "MSCs" in 2014. These reflect a broadening of research scope from cellular mechanisms to disease-specific applications.

The field reached a critical expansion point in 2015 with the emergence of its largest and most central theme, cluster #0 "neurodegeneration". That same year, cluster "diabetic retinopathy" also appeared. In 2016, the cluster "diabetes and cataracts" emerged, followed by clusters "tears" and "endophthalmitis" in 2017, indicating growing interest in clinical translation and non-invasive diagnostics. After that, no new thematic clusters have emerged; meanwhile, most clusters reached maturity by 2022. Accordingly, the knowledge base in this field has stabilized and matured.

#### Highly contributed cited references

The highly cited references serve as cornerstones in a research field, shaping its theoretical framework. Among top 10 cited publications (Table 5), three of the top five are United States-based studies, suggesting a geographic concentration of influential research output in this field. Meanwhile, the list includes six original research articles and four reviews, reflecting a balanced reliance on empirical evidence and conceptual analysis. Key reviews play pivotal roles in defining the fields scope and terminology. These include EVs roles in normal and diseased eyes (rank 4th) [14], and broader foundational reviews on EVs origin, biogenesis, and function by Kalluri & LeBleu (rank 5th) [3], Raposo & Stoorvogel (rank 7th) [15], and van Niel et al. (rank 10th) [16].

**Table 5:** Top 10 most cited references

First author (ref.)	Country	Year	Times cited	Type	Descriptions
Clotilde Théry [17]	France	2018	70	Article	MISEV2018 guidelines
Clotilde Théry [18]	France	2006	50	Article	Different methods for purifying and evaluating diverse EVs
Bean Mead [19]	USA	2017	48	Article	Exosomal miRNA Argo-2 from BMSCs protects RGCs
Mikael Klingeborn [14]	USA	2017	42	Review	The roles of EVs in normal and diseased eyes
Raghu Kalluri [3]	USA	2020	37	Review	Biogenesis, function, and biomedical applications of EVs
Hadi Valadi [21]	Sweden	2007	34	Article	Exosomal RNA and miRNA as intercellular mediators
Graça Raposo [15]	France	2013	33	Review	Characterization, formation, targeting, and function of EVs
Gum-Yong Kang [20]	South Korea	2014	30	Article	Exosomal proteins of AH as biomarkers for AMD
Ravand Samaeekia [22]	USA	2018	30	Article	corneal MSCs-EVs promoted corneal wound healing
Guillaume van Niel [16]	France	2018	30	Review	Origin, biogenesis, secretion, targeting, and fate of EVs

Argo-2: Argonaute-2. BMSCs: bone marrow MSCs. RGCs: retinal ganglion cells. AH: aqueous humor. AMD: age-related macular degeneration.

The most highly cited reference, titled *Minimal information for studies of extracellular vesicles 2018 (MISEV2018)*, published in 2018 by the International Society for Extracellular Vesicles (ISEV), provides a guideline for documenting EVs-associated functions, has received 70 citations, and has the highest average annual citation count (11.67) [17]. The second-ranked study (2006) focuses on EVs purification techniques, further reflecting the foundational importance of methodological rigor in the field [18]. The third-ranked study (2006) demonstrated that miRNA Argonaute-2 in bone marrow MSCs-derived EVs promotes RGC survival [19], marking the first application of bone marrow MSCs-EVs in vision science and thereby catalyzing subsequent therapeutic exploration. Other notable studies broaden clinical perspective. In particular, the identification of exosomal proteins (apo-A1, clusterin, cytokeratin 8, C3, PTGDS, cath D) as biomarkers for neovascular AMD (rank 8th) [20], the insights of exosomal mRNA and microRNA can be delivered to another cell and function in this new location (rank 6th) [21], and the therapeutic role of corneal MSCs-derived EVs via accelerating epithelial wound healing (rank 9th) [22].

#### Cited references with strongest citation bursts

Among the cited references with the strongest citation bursts, those with simultaneous bursts were grouped, and the most representative reference from each group received our primary focus. Accordingly, seven key publications stand out (Fig. 6B**)**, five of which also rank among the top 10 most cited references, indicating both sustained influence and temporal impact. Notably, the MISEV2018 [17] showed the second-highest citation burst (strength = 4.65); meanwhile, it was also the most highly cited. This underscores its pivotal role in standardizing EVs methodologies, improving reproducibility, and advancing EVs-based diagnostics and therapies in vision science.

The remaining two studies investigated EVs-related oxidative stress in RPE. Both exhibited the highest betweenness centrality and shared a sigma value of 0.27, underscoring their foundational role in retinal pathphysiology. Particularly, the first study, published in 2013, reported that oxidative stress alters EVs protein profiles involved in apoptosis, survival, and metabolic pathways, suggesting their involvement in pathophysiological events or potential as biomarkers [23]. The second study, published in 2016, exhibited the strongest (value = 4.92) and the longest (2017–2021) citation burst. This study revealed that oxidative stress increases EVs release from RPE, enriches them with vascular endothelial growth factor (VEGF) receptors and VEGF receptors´ mRNA, and promotes angiogenesis in endothelial cells [24].

### Research frontiers: analysis based on author keywords

#### Author keywords with high concurrency, citation bursts, and high frequency

**Fig. 7:**
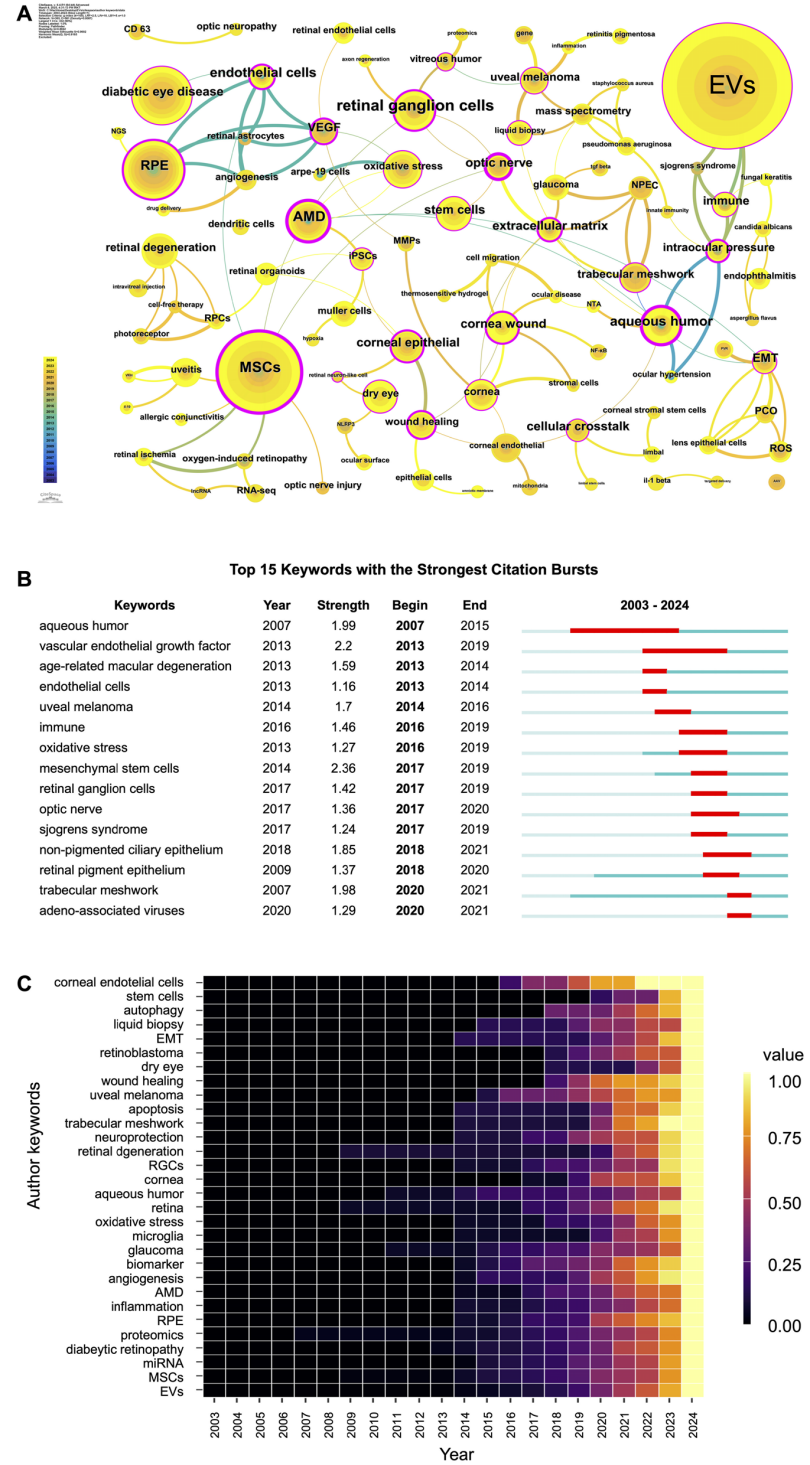
(**A**) The concurrence of high occurrence (≥ 2 times) and/or high centrality (≥ 0.1, marked with purple circle outsiside) of author keywords. Keyword size reflects frequency, while color and connecting lines indicate temporal appearance. **(B)** Top 15 author keywords with the strongest citation bursts. **(C)** The maturation over time of the top 30 most frequent author keywords.

The network of author keywords with high concurrency (≥ 2 concurrences) and/or high centrality (≥ 0.1) is illustrated (Fig. 7A). A total of seventeen author keywords concurrenced at least 10 times, with "EVs" being the most frequent keyword (187 times), followed by "MSCs" (73 times), "RPE" (43 times), "diabetic eye disease" (41 times). Other frequently co-occurring keywords (10 ≤ frequency < 20) include "retinal ganglion cells (RGCs)", "AMD", "oxidative stress", "aqueous humor", "retinal degeneration", "stem cells", "cornea wound", "dry eye", "cornea", "cornea epithelial", "TM", "uveal melanoma", and "VEGF". Notably, all these keywords except "retinal degeneration" also exhibit high betweenness centrality, suggesting their role as conceptual bridges between research subfields.

Furthermore, as shown in Fig. 7B, keyword "MSCs" had the strongest citation burst (strength = 2.36, 2017–2019), "aqueous humor" exhibited the earliest and longest citation burst (8 years, 2007–2015), while "VEGF" showed the second-longest citation burst period (6 years, 2013–2019). "Trabecular meshwork" and "RPE" first appeared in 2007 and 2009, respectively; however, their citation bursts were not observed until 2018 and 2020. This delay reflects a lag in concentrated research attention within this field. Additionally, 14 out of the 17 most frequently co-occurring keywords were also ranked among the top 30 high-frequency terms (Fig. 7C). All of them exhibited progressive maturation over time through 2024, with particularly accelerated development observed since 2020, which may be due to the growing sustained interest in this field.

#### Evolution trajectory of frontier themes

**Fig. 8:**
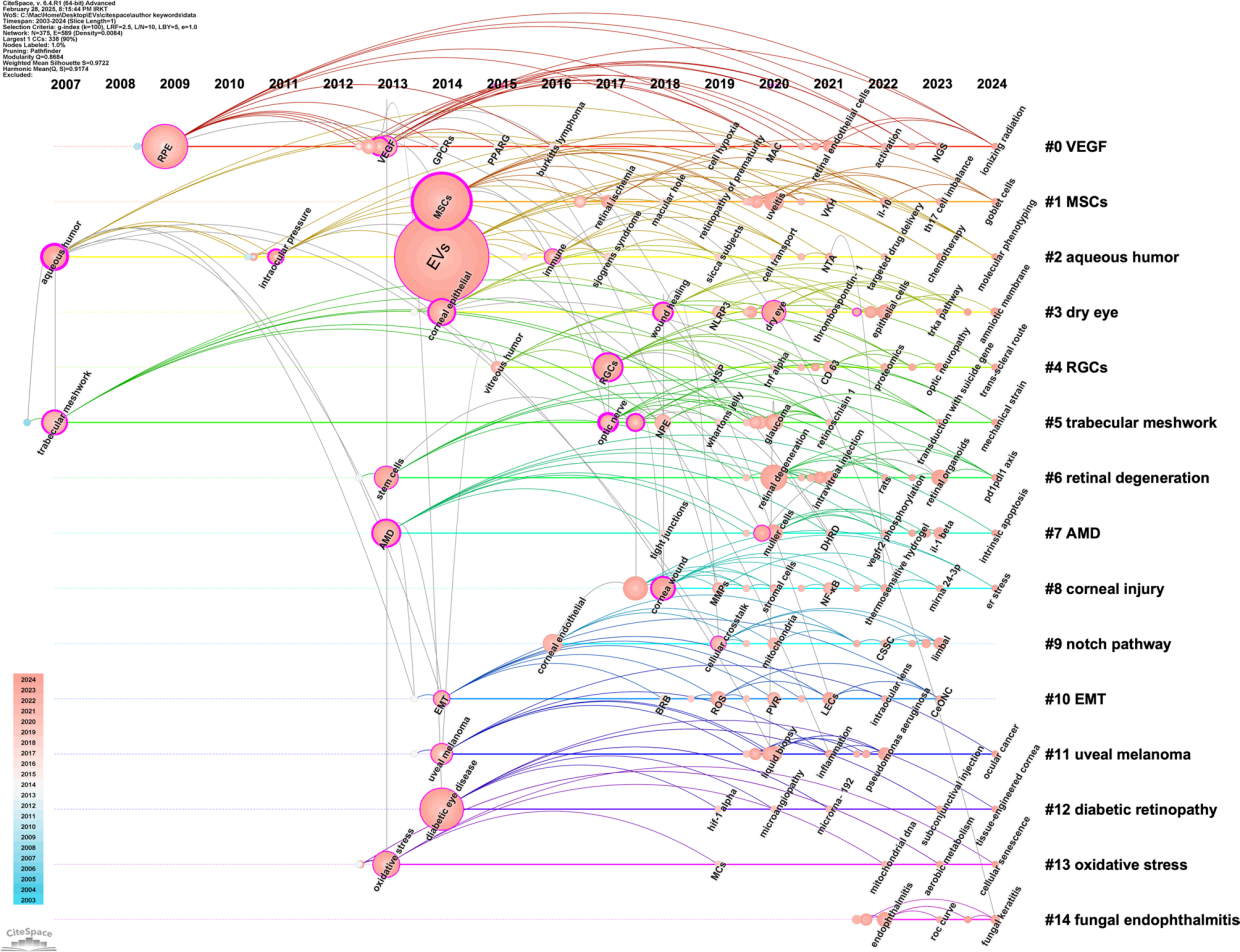
Timeline visualization of keyword co-occurrence cluster analysis on EVs in vision science

Between 2003 and 2024, a total of 15 thematic clusters were identified based on co-citation analysis using CiteSpace (Fig. 8). The earliest cluster emerged in 2007 and consisted of cluster 2 "Aqueous humor" and cluster 5 "Trabecular meshwork". The former primarily consists of "aqueous humor", "intraocular injection", "EVs", and "retinal endothelial cells". The latter is characterized by "TM", "optic nerve", "NPEC", and "glaucoma". These suggest an early focus on intraocular environments, the pathophysiology of intraocular pressure (IOP), and optic neuropathies.

The most prominent cluster, "VEGF" (cluster 0), was established in 2009. This cluster centers around "RPE", "VEGF", "angiogenesis", and "endothelial cells", underscoring a critical research focus on vascular signaling mechanisms. Following a three-year gap, the year 2013 marked a growing interest in retinal disorders, with the emergence of cluster 6 "retinal degeneration", cluster 7 "AMD", and cluster 13 "oxidative stress".

Cluster 1, labeled "MSCs", appeared in 2014 and was distinguished by "MSCs", "RNA-seq", "optic nerve injury", and "retinal ischemia", reflecting a growing interest in MSCs-based regenerative approaches and transcriptomic profiling. In the same year, four additional clusters emerged: cluster 3 "dry eye", cluster 11 "uveal melanoma", cluster 10 "epithelial mesenchymal transition (EMT)", and cluster 12 "diabetic retinopathy". They collectively indicate a thematic diversification toward both inflammatory and neoplastic ocular conditions, as well as molecular mechanisms such as EMT implicated in disease progression.

Cluster 4 "RGCs" emerged in 2015, reflecting a focus on the neuroprotection of RGCs. Subsequently, cluster 9 "notch pathway" appeared in 2016, indicating a rising interest in developmental and angiogenic signaling. After two year, cluster 8 "corneal injury" emerged in 2018 with core keywords "cornea" and "cornea wound", signaling a shift toward ocular surface repair and regenerative therapies. The most recent cluster "fungal endophthalmitis" (cluster 14) appeared in 2022 and remains in development, suggesting a nascent but growing focus on infectious etiologies and immune responses within intraocular environments.

Overall, thematic clustering of author keywords yielded a Modularity (Q) value of 0.8692 and a Silhouette (S) value of 0.9692, indicating well-separated, internally consistent, and thematically coherent clusters. Notably, cluster 9 "notch pathway" and cluster 13 "Oxidative stress" began gaining momentum in 2021 and 2022, respectively, while other clusters expanded as early as 2019 (suppl. Fig. 1). This reflects a sharp increase in research activity across diverse ocular topics related to EVs since 2019. Furthermore, except for the clusters "EMT" and "notch pathway", the remaining clusters are in a developmental phase, indicating emerging yet still maturing research directions in the field.

### Potential emerging topic in the future

**Fig. 9:**
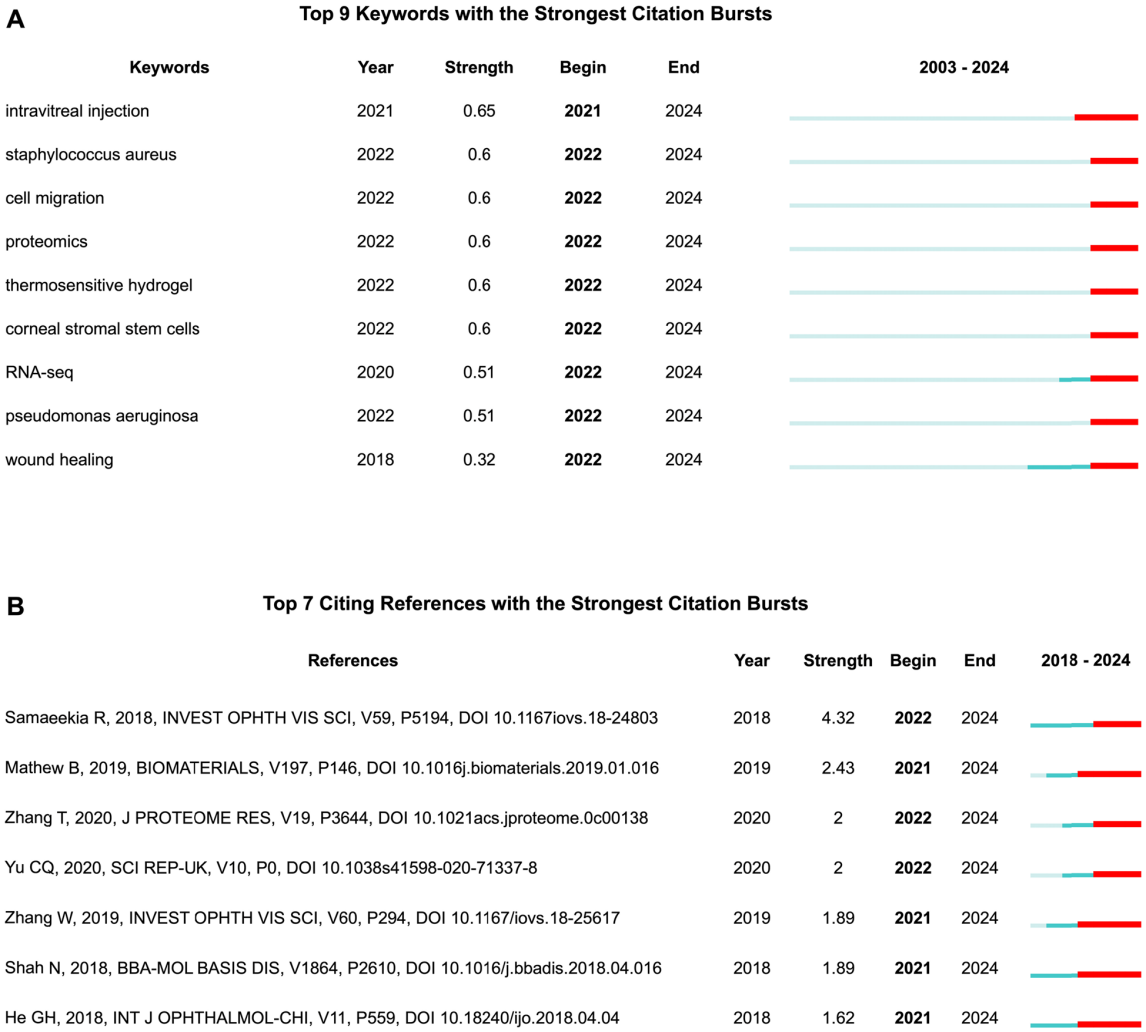
**(A)** Top 9 author Keywords with the strongest citation bursts through 2024. **(B)** Top 7 citing references with the strongest citation burst through 2024

Although the high citation rates of citing articles may shape emerging topics in the future [9], their citation counts span across all scientific disciplines rather than being specific to EVs research in vision science. In contrast, citation bursts in keywords and publications can offer more focused insights into emerging directions [25]. Therefore, we cross-validated emerging trends by integrating the top 10 highly citing papers with the citation bursts of both nine author keywords (Fig. 9A) and sevenciting papers (Fig. 9B), all of which sustained their citation bursts through 2024. It was identified that three of the top 10 highly citing articles were also identified within the citation bursts of citing papers.

Accordingly, studies on retinal neuroprotection [26] and retinal inflammation [27], along with the keywords "thermosensitive hydrogel" and "intraocular injection"(highest citation burst, strength value = 0.65), highlight growing interest in EVs-based drug delivery systems for targeted, sustained treatment of ocular posterior segment diseases such as retinal degeneration, diabetic retinopathy, and uveitis. The study on corneal wound healing [22], together with keywords "cell migration" , "corneal stromal stem cells", and "wound healing", suggests the potential of EVs as cell-free therapies for corneal wound healing. The keywords "Staphylococcus aureus" and "Pseudomonas aeruginosa" likely indicate emerging interest in EVs for bacterial keratitis and/or endophthalmitis. Meanwhile, the keywords "proteomics" and "RNA-seq" highlight advances in identifying bioactive molecules in EVs, supporting multi-omics-guided precision therapy in ocular disease.

## Discussion

This work presents a comprehensive scientometric analysis of original EVs research in vision science. Since 2023, its knowledge base has stabilized and matured, encompassing 13 key themes. Meanwhile, most research frontier clusters remain in early development. Research attention has sharply increased since 2020. This rapid expansion is in line with the broader growth of EVs research in medicine and bioengineering. Furthermore, it closely aligns with the NEI´s Strategic Plan titled *Vision for the Future (2021–2025)*, released in November 2021, which prioritizes EVs research as a key focus within regenerative medicine [5]. Due to the lack of similar studies conducted previously in this field, this discussion primarily explores the diverse sources of EVs, thematic clusters of research frontiers with a focus on EVs-associated ocular pathophysiology and therapeutic implications, potential diagnostic biomarkers, as well as bioengineering and delivery systems.

### Implicated EVs source in vision science

EVs originate from a wide range of sources but can be primarily classified into four main categories. Of note, among the 427 included publications, none reported the use of synthetic EVs mimics. The majority (~ 60%) are derived from ocular tissues or related cell types, such as conjunctival goblet cells, cornea, lens epithelial cells, limbus, TM, NPEC, aqueous humor, vitreous humor, and retina. Particularly, cornea-EVs play key roles in the wound healing of corneal injury. Lens epithelial cells-EVs are linked to ocular angiogenesis and posterior capsular opacification. Limbal cells-EVs are associated with diabetic corneas and the limbus itself, while TM- and NPEC-EVs are associated with glaucoma and IOP regulation. Vitreous humor-EVs are connected to diabetic retinopathy, proliferative vitreoretinopathy, uveal melanoma, pathological myopia, and fungal endophthalmitis. Retina-EVs are primarily involved in retinal degeneration, AMD, diabetic retinopathy, and retinal neovascularization. This predominance is likely attributed to the fact that the phenotype and function of EVs are closely associated with their parent cells [3], aligning with this work´s focus on EVs research in vision science.

**Fig. 10:**
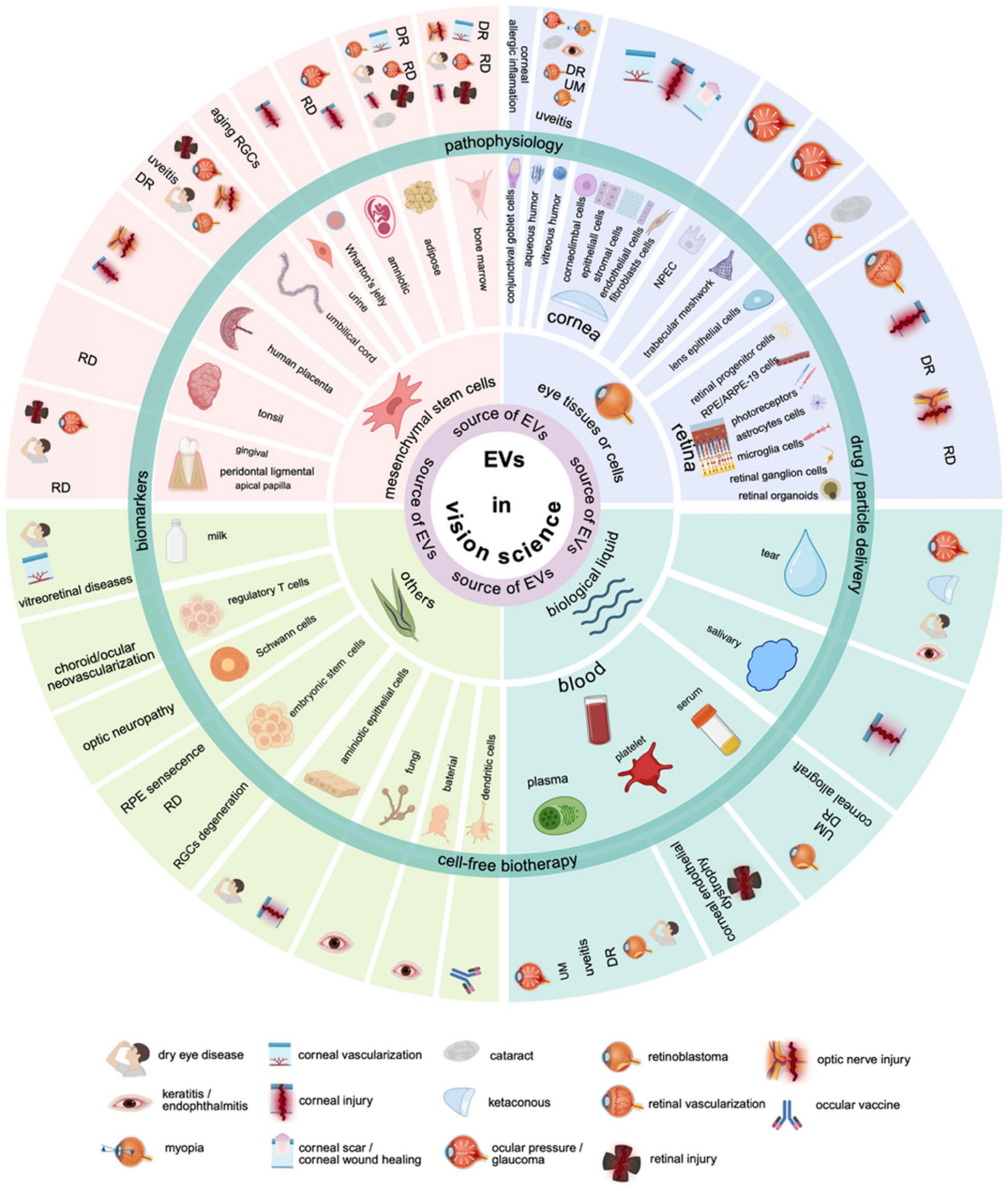
An overview of research conducted with EVs in the field of vision science. The four principal categories of EVs´ sources in visual science are indicated in the dark pink circle and their corresponding circular sector. The outside dark green circle and its circular sector represent the main implied functional roles of EVs in vision science. DR: diabetic retinopathy. EVs: extracellular vesicles. NPEC: non-pigmented epithelial cells. RD: retinal degeneration. RGCs: retinal ganglion cells. (Figure was created with Biorender.com)

MSCs represent approximately 25% of sources, while biological fluids, including blood (plasma, serum, and platelets), saliva, and tears, account for around 11%. The remaining 5% originates from other sources, such as embryonic stem cells, amniotic epithelial cells, milk, regulatory T cells, dendritic cells, Schwann cells, bacteria, and fungi. An overview of research conducted with EVs in the field of vision science is shown (Fig. 10).

### Thematic clusters: EVs in pathophysiology and therapeutic applications

#### Cluster 0: VEGF

VEGF is a key regulator of physiological angiogenesis. EVs may contribute to pathological angiogenesis. For instance, EVs derived from corneal fibroblasts induce corneal neovascularization via matrix metalloproteinases (MMP)14-mediated recruitment of MMP2, degradation of VEGFR1, and enhanced VEGFR2 activation by VEGFA [28–30]. Similarly, exosomal miR-155-5p of retinal microglia cells induces retinal neovascularization through the Irf1/miR-155-5p/Socs1 signaling axis [31].

In contrast, EVs exhibit anti-angiogenic effects. Studies have shown that EVs from lens epithelial cells can suppress ocular pathological neovascularization via exosomal miR-146a-5p [32] or by inhibiting microglial activation [33]. Exosomal miR-302a-3p from ARPE-19 cells-EVs directly targets VEGF-A mRNA to suppress oxidative stress-induced angiogenesis, while endostatin from retinal astrocytes-EVs inhibits laser-induced choroid neovascularization. These mechanisms may protect the eye from angiogenesis in diabetic retinopathy and wet AMD [34–37]. Furthermore, umbilical cord MSCs-EVs exhibit retinal anti-angiogenesis by downregulating HIF-1α and VEGF-A expression.

#### Cluster 1: Mesenchymal stem cells

MSCs represent the second most common EVs source in vision science and have been studied in both anterior and posterior ocular segments. MSCs-EVs are primarily obtained from bone marrow, adipose tissue, umbilical cord, and amniotic membrane. Meanwhile, MSCs-EVs can be obtained from urine, tonsils, and dental tissues (apical papilla, gingiva, and periodontal ligament). Such prominence is likely attributed to MSCs inherent immunomodulatory, regenerative, and neuroprotective capacity, along with a rich repertoire of bioactive molecules. Furthermore, MSCs-EVs offer a promising alternative to whole-cell therapy, demonstrating comparable or even superior biological activity to their parent cells [38].

#### Cluster 2: Aqueous humor

The size of aqueous humor-EVs may have different implications between normal and diseased eyes. Larger EVs (90–120 nm) are predominant in inflammatory ocular conditions, whereas exosomeres (~ 35 nm) are more prevalent in neovascular diseases [39]. Glaucomatous EVs are smaller than those in healthy controls [40], while no significant size differences are observed between myopia patients and controls [41].

While aqueous humor-EVs exhibit proliferative and regenerative properties that promote wound healing [42], they may contribute to ocular pathogenesis. Specifically, aqueous humor-EVs promote posterior capsule opacification by enhancing metastasis and EMT in human lens epithelial cells through exosomal miR-1246–mediated targeting of GSK-3β [43]. In diabetic cataracts, exosomal miR-29b reduces the viability of human lens epithelial cells by upregulating CACNA1C [44], while exosomal miR-551b downregulates CRYAA expression [45]. Additionally, exosomal C1QB EVs have been implicated in Vogt–Koyanagi–Harada and Behcets uveitis [46].

#### Cluster 3: Dry eye disease

MSCs represent the prominent EVs sources that have been demonstrated to have therapeutic potential for dry eye, which is achieved primarily by suppressing pro-inflammatory and/or upregulating anti-inflammatory factors. Mechanistically, umbilical cord MSCs-EVs inhibit dendritic cell-mediated Th17 immune responses [47], suppress TNF-α and IL-1β, and upregulate IL-10 and TGF-β [48], and modulate the IRAK1/TAB2/NF-κB pathway via exosomal miR-125b, let-7b, and miR-6873 [49]. Bone marrow MSCs-EVs reduce cell apoptosis and increase retinal neuron-like cell proliferation protein [50], reprogram pro-inflammatory M1 macrophages into immunosuppressive M2 via miR-204-mediated the IL-6/IL-6R/STAT3 pathway [51], and restore Treg/Th17 balance via the miR-21-5p/TLR4/MyD88/NF-κB pathway [52]. Adipose tissue MSCs (ADMSCs)-EVs suppress NLRP3 inflammasome and alleviate ocular surface damage [53] or downregulate Fbxw7 via exosomal miR-223-3p [54]. Dental periodontal ligament MSCs-EVs may protect conjunctival goblet cells from M1 macrophage-mediated inflammation [55]. Furthermore, EVs from human amniotic epithelial cells [56], M2 macrophages [57], and *Limosilactobacillus fermentum* HY7302 through the gut-eye axis [58] have been shown to restore corneal surface homeostasis in the treatment of dry eye. Nonetheless, EVs-associated pathophysiology in dry eye has not yet been reported.

#### Cluster 4: Retinal ganglion cells

The loss of RGCs and their axons, primarily due to traumatic (optic neuropathy) and degenerative (glaucoma) eye diseases, remains a leading cause of blindness. EVs exhibit RGCs neuroprotection and axogenic properties, which may be therapeutically relevant to RGCs-related disorders, as summarized in Table 6. These EVs include various subtypes derived from MSCs [59–68] as well as from vitreous humor [69], embryonic stem cells [70], Schwann cells [71], and retinal astrocyte cells [72]. Among these, MSCs represent the most prevalent source of EVs. Nonetheless, the majority of these EVs lack identified bioactive cargoes, and their underlying signaling mechanisms remain largely unexplored.

**Table 6:** Therapeutic potential of EVs in RGCs disorders

EVs source	Main cargo	Main function and/or associated pathway	Refs.
UMSCs		Inhibit RGCs apoptosis in glaucoma	[62]
Amniotic MSCs		Neuroprotective by anti-oxidative and recovering IOP in glaucoma	[63]
ADMSCs		Anti-apoptosis, boost neurotrophic factor secretion	[64]
WJ MSCs		Promote RGCs survival, regenerate axons, activate glia cells	[65, 66]
hPMSCs		Modulate mitochondrial homeostasis	[67]
urine MSCs		Enhance the survival and proliferation of aging retinal RGCs	[68]
BMSCs	miR-Arg-2	PI3K/AKT pathway, activate G-CSF-macrophage pathway	[19, 59–61]
Vitreous humor		Exhibit RGCs neuroprotection in NAAION	[69]
ESCs		Mitigate tauopathy in RGCs degeneration	[70]
Schwann cells		Activate the CREB pathway and regulate reactive gliosis	[71]
Astrocyte cells	Loxl1	Inhibit reactive astrocytosis	[72]

ADMSCs: adipose tissue-derived MSCs. BMSCs: bone marrow MSCs. CREB: cAMP-response element binding protein. ESCs: embryonic stem cells. hPMSCs: human placental MSCs. IOP: intraocular pressure. Loxl1: lysyl oxidase Like 1. miR-Arg-2: miR-Argonaute-2. NAAION: non-arteritic anterior ischemic optic neuropathy. UMSCs: umbilical cord MSCs. WJ: Wharton’s Jelly MSCs

#### Cluster 5: Trabecular meshwork

Exosomal proteins emilin-1, neuropilin-1, and glaucoma-causing myocilin are uniquely identified in TM-EVs [73, 74]. Exosomal miRNAs in TM-EVs regulate the communication between TM and Schlemms canal endothelial cells, with exosomal miR-7515 implicated in reprogramming the latter [75]. TM regulates IOP by draining aqueous humor; thus, EVs-based TM studies have focused on IOP or glaucoma. Studies found that glaucomatous TM-EVs express extracellular matrix (ECM)-related fibronectin and EDIL3, promoting glaucoma progression [76]. Dexamethasone and TGF-β2 can alter the cargo and properties of TM-EVs, causing pathological ECM accumulation in TM and promoting development of the primary open-angle glaucoma [77, 78].

However, EVs have demonstrated the capacity to reduce IOP. For instance, bone marrow MSCs-EVs protect TM cells from oxidative stress [79], whereas NPEC-EVs inhibit collagen type I fibrillogenesis in TM, a key process in ECM remodeling [80]. Anti-glaucoma drugs such as Timolol maleate, Brinzolamide, or Benzalkonium Cl affect the uptake of NPEC-EVs by TM cells [81]. Furthermore, Netarsudil enhances TM outflow by inducing phagocytosis and modulating EVs activity [82].

#### Cluster 6: Retinal degeneration

Retinal degeneration involves the progressive loss of retinal cells, particularly photoreceptor degeneration and dysfunction of the RPE. It has shown that RPE cells engulf rhodopsin-containing EVs secreted by degenerating rod photoreceptors, suggesting a potential mechanism for clearing unwanted proteins during photoreceptor degeneration in retinitis pigmentosa [83]. Additionally, in retinitis pigmentosa with a PDE6b mutation, poly (ADP-ribose) polymerase inhibition protects photoreceptors by modulating EVs activity in rod photoreceptor degeneration associated [84].

While retinal microglia cells-EVs exhibit pro-inflammatory properties under elevated IOP, contributing to glaucomatous retinal degeneration [85], human embryonic stem cell-EVs prevent degeneration by promoting Müller cell retro-differentiation via HSP90 signaling [86]. Notably, MSCs- and retina-EVs demonstrate significant therapeutic potential for retinal degeneration, as presented in Table 7. MSCs-EVs achieved such potential primarily by their retinal neuroprotective [87–90], antioxidant [91–93], anti-apoptotic [91], and anti-inflammatory [90] properties. Retina-EVs functioned in a source-specific mechanism [94–98]. However, the potential biocargoes of EVs and the elucidation of the signaling pathways need further exploration.

**Table 7:** MSCs- and retina-EVs therapeutic potential in retinal degeration

EVs source		Main cargo	Main function and associated pathway	Refs.
MSCs	BMSCs	miR-21	Photoreceptor protection	[88]
	ADMSCs	miR-222	Retinal tissue protection	[87]
			Anti-oxidative, anti-apoptosis. Nrf2 pathway	[91]
	UMSCs		Neuroprotection by upregulate GAP43	[89]
	Amniotic MSCs		Anti-oxidative. PI3K/Akt/FoxO3 pathway	[92]
	Tonsil MSCs		Anti-oxidative, protect RPE from all-trans-retinal-induced toxicity	[93]
	Apical MSCs		Neuroprotection and anti-inflammation by modulate Bax, Bcl-2, IL-10, IL-6	[90]
Retin	RPE		Construct a cytoprotective microenvironment	[95]
	Retinal organoids		Regulating fatty acid metabolism	[97]
			Photoreceptor protection, MAPK signaling pathway	[96]
	NPCs		Microglia inactivation	[94]
	hRPCs	#	Regulate autophagy, signal release, neuron death, and cell cycle	[98]

ADMSCs: adipose tissue-derived MSCs. BMSCs: bone marrow MSCs. GAP43: growth-associated protein 43. hRPCs: human retinal progenitor cells. NPCs: neural progenitor cells. RPE: retinal pigment epithelial. UMSCs: umbilical cord MSCs. #: exosomal miR-21-5p, let-7i-5p, miR-100-5p, miR-148a-5p, and miR-151a-3p

#### Cluster 7: Age-related macular degeneration

AMD, primarily caused by RPE dysfunction, is the leading cause of vision loss in the elderly. Meanwhile, oxidative stress-induced RPE dysfunction is a key contributor to early dry AMD. As such, RPE-EVs have been extensively studied. Pathogenically, oxidative stress alters RPE-EVs phosphoproteins [23] and miRNA profiles (upregulates miR-7-5p, downregulates miR-183, miR-125b-5p, miR-125a-5p, miR-128-3p) [99, 100]. Oxidative RPE-EVs enhance EVs release from aged RPE cells, promote drusen formation, and exacerbate oxidative stress damage to RPE. This damage is mediated by upregulating the cyclin-dependent kinase inhibitors p15 and p21, as well as promoting senescence and interrupting phagocytic activity of RPE cells [101, 102]. Furthermore, oxidative RPE-EVs activate retinal microglia via ZBP1 signaling [103], enhance NLRP3 inflammasome activation under photooxidative blue-light stimulation [104], promote calpain-2–mediated autophagy-lysosomal dysfunction and apoptosis [105], and induce inflammation through lncRNA CYLD-AS1 via modulation of the NRF2/NF-κB pathway [106]. Of note, AMD-affected RPE-EVs induce AMD-like phenotypes in neighboring RPE and retinal cells, thereby exacerbating disease progression [107]. 

Nevertheless, normal RPE-EVs miR-182, miR-183, and miR-122 contribute to RPE homeostasis and function in a polarized manner [100], whereas αB crystallin, miR-302, and miR-122 exhibit anti-inflammatory and anti-apoptotic effects [108, 109]. Additionally, embryonic stem cells-EVs may reverse the senescence of RPE through the p38MAPK pathway [110], and ADMSCs-EVs protect RPE cells from oxidative damage via the Nrf2/Kepa1 pathway [111]. Collectively, these EVs offer preventative and protective therapy in AMD.

#### Cluster 8: Cornea injury

Cornea-EVs and MSCs-EVs exhibit robust therapeutic potential in corneal injuries and associated processes, such as scarring, inflammation, and wound healing. Mechanistically, cornea-EVs from epithelial cells, myofibroblasts, and endothelial cells mediate corneal interlayer communication and exert therapeutic effects via regulating HSP27, STAT, β-catenin, GSK-3β, and p38 pathways, as well as the presence of ECM-related fibronectin and thrombospondin-1 (inhibits hypoxia-induced paraptosis) [112–117]. Corneal stromal stem cells-EVs regenerate transparent corneal stromal tissue via anti-inflammatory and anti-fibrotic miR-29a and miR-381-5p [22, 118, 119].

Bone marrow MSCs-EVs demonstrate anti-inflammation, anti-angiogenesis, and anti-apoptosis effects via the NF-κB c-Rel, p44/42 MAPK, PI3K/AKT/mTOR, and mTOR/NF-κB/IL-1β pathways [120–126]. Similarly, umbilical cord MSCs-EVs target the PTEN/PI3K/Akt pathway via miR-21 [127]. Amniotic MSC-EVs trigger DNMT1-mediated miR-33 promoter hypermethylation in corneal epithelial cells [128]. ADMSCs-EVs deliver miR-19a to suppress HIPK2 and myofibroblast differentiation [129, 130]. Furthermore, EVs from retinal organoids [131], salivary [132], and amniotic epithelial cells [133] exert anti-inflammatory, regenerative, and ECM-supportive microenvironments, respectively.

#### Cluster 9: Notch pathway

There are two studies about the notch pathway. One reveals that in retinal degeneration models, bEnd.3 endothelial cell-derived EVs delivering miR-449a inhibitors promote human retinal progenitor cells (hRPCs) survival and reduce apoptosis more effectively than miR-449a inhibition alone by upregulating Notch signaling [134]. Similarly, corneal stromal stem cell-derived exosomal miRNAs, including has-miR-663b, has-miR-16-5p, and has-miR-1290, enhance the proliferation and stemness of limbal epithelial stem cells, offering potential treatments for corneal epithelial disorders [135].

#### Cluster 10: Epithelial mesenchymal transition

EVs may promote or inhibit the EMT, which is a crucial biological process for embryogenesis, wound healing, and malignant progression. Particularly, under the proliferative vitreoretinopathy microenvironment, RPE-EVs promote EMT in recipient cells through exosomal R345W-Fibulin-3 binding to TGF-β [136] or via exosomal miR-543, serving as the key mediators of cellular communication [137]. Lens epithelial cell-EVs contribute to the reactive oxygen species-induced lens EMT, preventing posterior capsule opacification [138]. In contrast, exosomal miR-4488 and miR-1273g-5p inhibit TGF-β2-stimulated EMT in ARPE-19 cells by targeting ATP-binding cassette A4 [139]. Meanwhile, exosomal miR-27b of umbilical cord MSCs suppresses EMT by targeting HOXC6, thereby attenuating subretinal fibrosis [140].

#### Cluster 11: Uveal melanoma

Uveal melanoma is the most common form of adult eye cancer. Uveal melanoma-derived EVs promote metastasis by modulating immune responses through macrophage migration inhibitory factors and preparing the metastatic niche [141], or by releasing into the liver circulation in metastatic uveal melanoma [142]. Studies revealed that the concentration of aqueous humor-EVs is elevated in uveal melanoma patients compared to those receiving brachytherapy [143]. Additionally, post-brachytherapy uveal melanoma samples show increased proportions of CD63/81 + and CD9/63/81 + EVs [144]. Proteomic and miRNA analyses of EVs isolated from aqueous humor, vitreous humor, and plasma of uveal melanoma patients have revealed consistent profiles, highlighting blood as a noninvasive liquid biopsy for uveal melanoma monitoring [143]. Furthermore, modified EVs carrying suicide genes show potential for targeted uveal melanoma therapy [145], and Ergolide exhibits anti-cancer effects on metastatic uveal melanoma cells by altering cellular and exosomal proteomes [146].

#### Cluster 12: Diabetic retinopathy

Diabetic retinopathy is the leading cause of vision loss among individuals with diabetes, primarily affecting retinal blood vessels. Pathogenically, elevated MMP2 levels in bone marrow MSCs- and ADMSCs-EVs induce vessel destabilization in early diabetic retinopathy [147]. RPE-derived exosomal miR-202-5p contributes to proliferative diabetic retinopathy by activating the TGF-β/Smad pathway [148]. Müller glia-derived exosomal miR-9-3p promotes angiogenesis by downregulating the sphingosine-1-phosphate receptor 1 [149]. Additionally, plasma-EVs induce retinal vascular damage through the deposition of the membrane attack complex [150, 151]. Whereas platelet-rich plasma-derived EVs may promote retinal fibrogenesis by activating the YAP pathway, thereby contributing to diabetic retinopathy development [152].

Of note, umbilical cord MSCs-EVs [153–158], bone marrow MSCs-EVs [159–163], and ADMSCs-EVs [164] have been shown to exhibit therapeutic potential in diabetic retinopathy. This potential is primarily attributed to their anti-inflammatory, anti-fibrotic, anti-angiogenic, and antioxidant properties, as summarized in Table 8. Furthermore, RPE can phagocytose and degrade photoreceptor-derived EVs, thereby delaying diabetes-induced photoreceptor cell degeneration [165].

**Table 8:** MSCs-EVs therapeutic potential in diabetic retinopathy

EVs source	Main cargo	Main function and associated pathway	Refs.
UMSCs	miR-22-3p	Suppress NLRP3 inflammasome activation	[156]
	miR-17-3p	Anti-inflammation, anti-oxidative, targeting STAT1	[157]
	miR-5068, miR-10228	Anti-apoptosis, anti-inflammation, anti-angiogenesis, HIF-1α/EZH2/PGC-1α pathway	[155]
	miR-18b	Anti-inflammation, anti-apoptotic, MAPK/NF-κB pathway	[153, 154]
	NEDD4	Anti-oxidative, anti-apoptosis, PTEN/AKT/Nrf2 pathway	[158]
BMSCs	miR-486-3p, miR-34a-5p, lncRNA SNHG7	Anti-inflammation, anti-angiogenesis. Pathway: TLR4/NF-κB, miR-34a-5p/XBP1, Wnt/β-catenin	[159–163]
ADMSCs	miR-192	Anti-inflammation, anti-angiogenesis, targeting ITGA1	[164]

ADMSCs: adipose tissue-derived MSCs. BMSCs: bone marrow MSCs. NEDD4: neural precursor cell expressed developmentally down-regulated protein 4. SNHG7: small nucleolar RNA host gene 7. UMSCs: umbilical cord MSCs

#### Cluster 13: Oxidative stress

Under oxidative stress conditions, EVs derived from RPE/ARPE-19 cells induce inflammation and apoptosis in normal RPE cells through the Apaf1/caspase-9 signaling axis [166] or contribute to the pathogenesis of AMD as previously reported [23, 99–104, 106]. Notably, the remarkable decrease in exosomal miR-183 released from apical RPE may serve as an indicator of RPE stress [100]. In contrast, EVs have been shown to exhibit significant therapeutic potential against oxidative damage. For instance, bone marrow MSCs-EVs protect TM cells [79], umbilical cord MSCs-EVs protect RPE or retinal cells [164–166], and ADMSCs-EVs help prevent cataracts [167]. Similarly, NPEC-EVs regulate IOP in glaucomatous conditions [168–171], while lens epithelial cell-EVs target age-related cataract via exosomal miR-222-3p [172]. Furthermore, MSCs-EVs alleviate oxidative stress-induced in retinal degeneration [91, 92], AMD [111], and diabetic retinopathy [157–163] as previously mentioned.

#### Cluster 14: Fungal endophthalmitis

Endophthalmitis is a vision-threatening complication that can occur following intraocular surgery or penetrating ocular injury. Proteomic analysis revealed that EVs cargo might contribute to its pathogenesis. Particularly, in *Staphylococcus aureus* and *Pseudomonas aeruginosa* endophthalmitis, exosomal proteins such as annexin A5, cathepsin D, C5a, Tenascin, calpain-2, caveolin-1, and caveolin-2 play major pathogenic roles [173–175]. In *Aspergillus flavus* and *Candida Albicans* endophthalmitis, EVs proteins, including Annexin 6, calpain-2, sorcin, fibroblast growth factor 2, Rab GDP dissociation inhibitor, and S100-A6, may contribute to the pathogenesis by activating MAPK, HIF-1, and PI3K-AKT immune signaling pathways [176].

### EVs biocargoes as potential biomarkers for ocular diseases

EVs play a pivotal role in cellular communication through their diverse biocargoes, making them promising biomarkers for ocular diseases (Table 9). They serve as a potential source of diagnosis include: dry eye [177], dry or wet AMD [20, 178], glaucoma [40, 179–182], pseudoexfoliation glaucoma [183], myopia [41], high myopia [184], retinoblastoma [185–189], uveal melanoma [190, 191], diabetic retinopathy [192–199], bacterial or fungal endophthalmitis [200, 201], diabetic keratopathy [202], central serous chorioretinopathy [203], myopic maculopathy deterioration [204], uveitis and scleritis [205], and diabetic macular edema [206]. These biomarkers primarily consist of exosomal miRNAs, proteins, and other molecules. They are mainly derived EVs isolated from biofluids such as aqueous humor, vitreous humor, plasma, serum, and tear fluid, which can be obtained through non-invasive or minimally invasive procedures.

**Table 9:** Potential EVs biomarkers for ocular diseases

EVs source	Representative cargo	Biomarker application	Refs.
Aqueous humor	apo-A1, clusterin, cytokeratin 8, C3, PTGDS, cath D	Wet AMD	[20]
	↓ SERPINA1, ↓AZGP1	Anti-VEGF progression of AMD	[210]
	GAS6, SPP1, RAD23B, PD-L1	Glaucoma	[179–181]
	Syntenin	Pseudoexfoliation glaucoma	[183]
	STT3B, miR-27, miR-199, miR-23, miR-130b	Primary open-angle glaucoma	[40, 182]
	has-miR-582-3p, −17-5p, −885-3p, −19b-3p, −450b-5p	Myopia	[41]
	transthyretin, hemopexin, cystain-C, Apo-H, Apo A-IV, Apo A-II, HRG, DKK3	High myopia	[184]
	CD133, enriched mono-CD63 +, ↑ CD63/81 +	Retinoblastoma	[185–187]
	miR-184	Central serous chorioretinopathy	[203]
Vitreous humor	miR-143-3p, miR-145-5p	Myopic maculopathy deterioration	[204]
	C8 alpha, calpain-2	Bacterial endophthalmitis	[200]
	Aquaporin-5	Fungal endophthalmitis	[201]
	LDHA, ficolin 3, miR-125, miR-21, apo-B, apo-M	Diabetic retinopathy	[197, 198]
	miR-146a	Uveal melanoma	[190]
	miR-494-3p	Dry AMD	[178]
Plasma	miR-15a, miR-30b, miR-431-5p, TNFAIP8, miR-21-3p, miR-30b-5p, miR-150-5p	Diabetic retinopathy	[192–196]
	flotillin 2	Diabetic keratopathy	[202]
	miR-5787, miR-6732-5p	Retinoblastoma	[188]
	complement factor H-related protein 5	Therapeutic progression of NMO	[208]
	miR-29s	Therapeutic progression of glaucoma	[209]
	SAA1, orosomucoid-1, complement C9, CRP	Uveitis and scleritis	[205]
	miR-144-3p	Thyroid-associated orbitopathy	[211]
Serum	circRNA MKLN1	Diabetic retinopathy	[199]
	miR-146a	Uveal melanoma	[190]
	miR-377-3p	Diabetic macular edema	[206]
	IL-2, IL-22, IL-12(p40), Pentraxin-3, TNFSF13B, TNFSF8	Early uveal melanoma metastasis detection	[207]
	hsa-miR-191-5p, −223-3p, −483-5p, −203a	Uveal melanoma	[191]
Tear	↓ mRNA SCNM1, ↑ miR-130b	Dry eye disease	[177]
RB cells	ABI2, GSTM, neurocan, ATP6V1C1, SNAP25, PPP2R2A, UBX domain protein 4	Retinoblastoma	[189]
RBVS cells	LRP-1, osteoglycin	Retinoblastoma vitreous seeds	

ABI2: Abl interactor 2. AMD: age-related macular degeneration. Apo.: Apolipoprotein. ATP6V1C1: V-type proton ATPase subunit C 1. AZGP1: zinc-alpha-2-glycoprotein. Cath D: cathepsin D. CRP: c-reactive protein. DKK3: Dickkopf-related protein 3. GAS6: Growth Arrest-specific 6. GSTM: glutathione s-transferase Mu. HRG: Histidine-rich glycoprotein. LDHA: lactate dehydrogenase A. LRP-1: low-density lipoprotein (LDL) receptor-related protein. NMO: neuromyelitis optica. PPP2R2A: serine/threonine-protein phosphatase 2A. PTGDS: prostaglandin D2 synthase. RB: retinoblastoma. RBVS: RB vitreous seedings cells. SAA1: serum amyloid A-1 protein. SCNM1: sodium channel modifier 1. SERPINA1: Serpin Family A Member 1. SNAP25: synaptosome Associated protein 25. SPP1: Secreted Phosphoprotein 1. TNFAIP8: Tumor necrosis factor-α-induced protein 8. TNFSF: Tumor necrosis factor superfamily. VEGF: vascular endothelial growth factor

Of note, serum exosomal protein including IL-2, IL-22, IL-12(p40), Pentraxin-3, TNFSF13B, and TNFSF8 can be applied for early detection of uveal melanoma progression into metastatic disease [207]. Meanwhile, EVs biomarkers can monitor therapeutic responses, for instance, plasma-EVs CHFR5 in neuromyelitis optica [208] while miR-29 s in glaucoma [209], and relevant reduced levels of SERPINA1 and AZGP1 in aqueous humor following anti-VEGF treatment in AMD [210]. Nevertheless, no universal biomarkers have been identified.

### Comparative evaluation of EVs from diverse sources

Given the diversity of EVs sources, a total of 12 studies have been conducted to compare the physical and functional properties of EVs in vision science (Table 10). EVs from diverse sources exhibit both shared and unique characteristics [42, 97, 212–215]. Common effects across ADMSCs-, bone marrow MSCs-, and umbilical cord MSCs-EVs exhibit retinal neuroprotective effects [64, 216]. Similar physical properties have been shown between human induced pluripotent stem cells-EVs and umbilical cord MSCs-EVs [215], between bone marrow MSCs-EVs and corneal epithelial cells-EVs [213]. Consistent proteomic profiles in aqueous humor-, vitreous humor-, and plasma-EVs from uveal melanoma patients highlight blood as a noninvasive liquid biopsy for uveal melanoma monitoring [143].

**Table 10:** Comparison of EVs derived from different sources

	**Compared EVs Source**	**Characteristics**	**Refs.**
**Similarities**	ADMSCs, BMSCs	Reduce RGCs injury (↓apoptosis, ↑neurotrophic factor)	[64]
	ADMSCs, UMSCs	↓Inflammation & apoptosis in laser-reduced retinal injury	[216]
	AH, VH, Plasma	Similar protein profiles in uveal melanoma	[143]
	iPSCs, UMSCs	Morphology (physical properties)	[215]
	BMSCs, HCEC	Morphology (physical properties)	[213]
**Differences/Unique Advantages​​**	hESCs, hRPCs	hESCs-EVs: ↑proliferation (enriched in angiogenesis- and cell cycle- related proteins); hRPCs-EVs: enriched proteins of immune modulation/retinal development/lipid metabolism	[97]
	ADMSCs, ARPE-19 cells	ADMSCs-EVs: 11 upregulated, 34 downregulated miRNAs	[91]
	BMSCs, AH	AH-EVs:​​ stronger wound repair in vitro wound healing assay on HaCaT cells	[42]
	BMSCs, Serum	BMSCs-EVs: better for corneal endothelial dystrophy (Akt↑, ER stress↓)	[214]
	iPSCs, UMSCs	iPSCs-EVs: better for corneal wound healing	[215]
	BMSCs, HCEC	BMSCs-EVs: stronger corneal healing, anti-inflammation, anti-apoptosis	[213]
	ADMSCs, HCEC	ADMSCs-EVs: distinct miRNA profiles; stronger endothelial regeneration	[212]

ADMSCs: adipose tissue-derived MSCs. AH: aqueous humor. BMSCs: bone marrow MSCs. ER: endoplasmic reticulum. HCEC: human corneal epithelial cells. hESCs: human embryonic stem cells. hRPCs: human retinal progenitor cells. iPSCs: human induced pluripotent stem cells. VH: vitreous humor. UMSCs: umbilical cord MSCs

However, EVs unique properties are also evident. For instance, proteomic profiles between embryonic stem cells-EVs and hRPCs-EVs [97], miRNA profiles among ADMSCs-, ARPE-19 cells-, and corneal epithelial cells-EVs [91, 212]. Particularly, hRPCs-EVs contain unique protein of lipid metabolism regulation, making them as a promise for the treatment of lipid-related RPE disorders [97]. Bone marrow MSCs-EVs or ADMSCs-EVs demonstrate superior therapeutic effects than corneal epithelial cells-EVs in treating cornea-related disorders [212, 213]. Taken together, there are no comparative studies evaluating the efficacy of EVs from all diverse sources for specific ocular conditions, such as inflammation, neovascularization, and fibrosis. The shortage of such comparative studies makes it challenging to definitively determine which EVs source holds the greatest therapeutic potential, since they are likely source-specific therapeutic relevance.

### Bioengineering and EVs delivery system

Despite their inherent advantages in delivering bioactive molecules, natural EVs still face three major limitations as therapeutic vectors: limited targeting specificity, poor circulation kinetics, and restricted therapeutic content [217, 218]. To address these challenges, approximately 10% of studies have explored bioengineering strategies to enhance EVs targeting in ocular diseases, leveraging EVs versatile, customizable, and biocompatible properties (Table 11). The table is based on EVs sources, ocular disease types, and loading strategies. Endogenous loading involves transfecting parental cells prior to EV isolation [124, 127, 165, 219–232], whereas exogenous loading strategies entail direct modification of EVs [233–259].

**Table 11:** Bioengineering and EVs delivery system in vision science

EVs source		Modification		Application	Refs.
		Cargo/agent	Encapsulation method		
MSCs	iPSCs-MSCs	Thermosensitive hydrogel	Constant stirring	Corneal injury/complications	[238]
	UMSCs	miR-21	Transfection		[127]
	ADMSCs	miR 24-3p, DEGMA-HA hydrogels	Electroporation		[240]
	BMSCs	miR-29b-3p	Transfection		[124]
		PLGA electrospinning nanofibers scaffold	Incubation		[239]
		siRNA c-Rel	Transfection		[126]
		TNF-α	Stimulated	Glaucoma, RGC damage/degeneration	[219, 220]
	ADMSCs	Nicotinamide	Extrusion		[242]
	UMSCs	miR-22 overexpressing	Lentiviral transduction		[221]
		Rapamycin, IL-10	Sonication	Uveitis	[243, 244]
		Over-expressing CD73	Lentiviral transduction		[245]
		IL-10 overexpressing	Lentiviral transduction		[222]
	BMSCs	TAT-peptide	Transfection		[223]
		Cerium oxide	Incubation		[246]
		IL-23	Primed	Retinal degeneration	[224]
	UMSCs	RGD peptide, antagonist of IL-1 receptor	Incubation- extrusion		[247]
		miR-5068, miR-10228	Electroporation	Diabetic retinopathy	[155]
	BMSCs	Anti-VEGF drug bevacizumab	Freeze–thaw cycle, incubation, sonication		[248]
		Degradable PLGA microcapsules	Emulsion and solvent extraction, incubation	Vitreoretinal diseases	[241]
		Cerium oxide	Incubation	Dry eye disease	[249]
		Gold nanoparticles reduced ascorbic acid	Incubation		[250]
		IFNγ	Stimulated	Retinitis pigmentosa	[225]
		Overexpressing miR-424	Lentiviral transduction	Visual deficits in mTBI	[259]
				Retinal ischemic	[252]
		Liproxstatin-1 hydrogel	Incubation		[253]
	UMSCs	PEDF	Sonication	Retinal vascularization	[233]
ocular cells	LECs	Drug: Doxorubicin	Electroporation	PCO	[257]
	Retinal multicells	ASL	Sonication	CNV	[234]
	RPE cells	Dual drug: Daunorubicin and Dexamethasone	Electroporation	PVR	[255]
	RGCs	PACAP38	Via anchor peptide CP05	TON	[258]
	661w cells	Thioredoxin	Transfection	Diabetic retinopathy	[165]
	Y97 cells	Piperlongumine	Incubation	Metastatic retinoblastoma	[227]
Other sources	293 cells	AAV2, AAV2-RS1- ZsGreen + plasmid	Transduction	Ocular gene therapy	[229–231]
		Overexpressing miR-146a	Transfection	Retinitis pigmentosa	[226]
	Microglia	Thrombospondin 1	Knockout	Retinal vascularization	[228]
	M1 macrophages	EGF	Conditioned medium	Ocular surface anti-inflammatory	[232]
	Tregs	Anti-VEGF antibody	Via a peptide linker	CNV	[237]
		Degradable polymeric microcapsules	Emulsion and solvent extraction, incubation	Vitreoretinal diseases	[241]
	Milk	Lutein	Ultrasonic	Dry eye disease	[251]
		CARPs	PEG2000- lipid	Vitreoretinal diseases	[254]
		Drug: Ranibizumab	Saponin-assisted loading	Corneal vascularization	[235]
	Breast cancer cells	Drug: Doxorubicin	Unmodification	Retinoblastoma	[256]
	HUVECs	Peptide KV11	anchoring peptide CP05	Retinal vascularization	[236]
	bEnd.3 cells	miR-449a inhibitor	Transfection	Retinal degeneration	[134]
Plasma		miR-29b-3p	Electroporation	Glaucoma	[209]

ADMSCs: adipose tissue-derived MSCs. ASL: membrane Anchor (BODIPY), Spacer (PEG), and targeting Ligands (cyclic RGD peptide). BMSCs: bone marrow MSCs. CARPs: cationic arginine-rich peptides. CNV: choroidal neovascularization. EGF: Epidermal Growth Factor. HUVECs: Human umbilical vascular endothelial cells. iPSCs: human induced pluripotent stem cells. LECs: lens epithelial cells. mTBI: visual deficits in a mild traumatic brain injury. PACAP38: Pituitary Adenylate cyclase-activating polypeptide 38. PCO: Posterior capsule opacification. PLGA: poly(lactic-co-glycolic acid). PVR: proliferative vitreoretinopathy. RGCs: retinal ganglion cells. RPE: retinal pigment epithelial. TAT: trans-activator of transcription peptide. TON: traumatic optic neuropathy. Tregs: Regulatory T cells. UMSCs: umbilical cord MSCs

Engineering EVs sources include MSCs, ocular cells, plasma, and others. Among them, MSCs are the most bioengineered EVs sources, particularly from bone marrow MSCs and umbilical cord MSCs. Encapsulated payloads primarily consist of miRNAs (e.g., miR-21, miR-22, miR-24-3p, miR-29b-3p, miR-424, miR-5068, miR-10228), anti-inflammatory cytokines (e.g., IL-10, IL-23, TNF-α, IFNγ), peptides (e.g., TAT, RGD), small molecules (e.g., rapamycin, cerium oxide, nicotinamide), and proteins. Given the heterogeneity of molecular diversity and to accommodate therapeutic purposes, various bioengineering approaches have been applied to modify EVs, including transfection, lentiviral transduction, electroporation, sonication, saponin-assisted loading, extrusion, incubation, and freeze–thaw. Additionally, to enhance EVs stability and targeted delivery, biomaterial platforms have been integrated into EVs modifications, for instance, hydrogels [238, 240, 253], nanofibrous scaffolds [239], and PLGA (poly(lactic-co-glycolic acid)) microcapsules [241].

Bioengineered EVs exhibited significant translational promise in vision science, with applications targeting corneal injuries [124, 126, 127, 238–240], pathogenic vascularization [228, 233–237], uveitis [222, 223, 243–246], glaucoma [209, 219–221, 242], retinal degeneration [134, 224, 247], diabetic retinopathy [155, 165, 248], dry eye [249–251], vitreoretinal diseases [241, 254, 255], as well as retinoblastoma [227, 256], retinitis pigmentosa [225, 226], retinal ischemia [252, 253], ocular gene therapy [229–231], posterior capsule opacification [257], traumatic optic neuropathy [258]. However, clinical translation remains to be validated by their immunogenicity and potential side effects.

### Barriers to clinical translation

Despite tremendous gains in developing EVs research in vision science, most of them remain at the preclinical stage. Up to the present, two related clinical studies have been carried out. One study revealed that bone marrow MSCs-EVs alleviated graft-versus-host disease-associated dry eye disease and highlighted miR-204 as a potential therapeutic agent [51]. The other study indicated that human umbilical cord MSCs-EVs promoted both functional and anatomical recovery, thereby improving visual outcomes after surgery for refractory macular holes [260]. Under such circumstances, a search of the Cochrane Library was performed, identifying only five registered clinical trials, all of which involved MSCs-derived EVs.

 These include one trial on retinitis pigmentosa, one on corneal epithelial defects, and three on dry eye (Suppl. Table 6). However, the clinical efficacy of these therapies remains to be fully established. There are several potential challenges that may hinder the clinical translation of EVs in vision science. First, although around 15% of studies focus on exosomal RNA-related vision science, it remains a lack of standardization in EVs-derived RNA sequencing technologies and bioinformatic workflows, which are essential for minimizing pre-analytical variability and ensuring reliable downstream data [261]. Second, the proteomic profiling of EVs typically requires large sample volumes (>200 μL), making routine clinical implementation challenging. While EVs from three-dimensional cultures offer higher yields and enhanced biological activity [262], this approach has yet to gain widespread adoption, with only two studies, both of which are cornea-related, reporting its application [114, 135]. Third, the bulk EVs analysis compromises diagnostic and therapeutic precision due to the intrinsic heterogeneity of EVs subpopulations. Fourth, the specialized instrumentation required for EV isolation is not widely accessible, making the large-scale production of clinical-grade EVs with consistent quality and preserved bioactivity both technically and financially challenging [218, 261, 263]. As such, further efforts are warranted to advance the clinical translation of EVs.

### Current EVs research limitations, gaps, and possible future opportunities 

Beyond the shortage of clinical data, the current EVs research in vision science remains limited to some extent, as presented in Suppl. Table 7, which provides a thematic cluster-based summary, along with potential gaps and future opportunities. As such, to date, EVs-associated pathophysiological mechanisms have not been reported in dry eye, corneal injury complications, or pre-metastatic uveal melanoma. At the same time, there is a limited understanding of their roles in pathological angiogenesis, wet AMD, retinal degeneration, and endophthalmitis. Gaps remain in characterizing potential biomarkers and tracking EVs cargoes variation in early/late stages of progressive diseases such as diabetic retinopathy, AMD, and retinal degeneration. Despite these challenges, engineering EVs represents promising future opportunities, aiming to deliver therapeutic cargoes by selectively targeting ocular tissues, corneal layers, or retinal neurons, as well as by improving sustained release of encapsulated payloads. 

### Emerging single-vesicle analysis

Recent developments in single-vesicle or single-cell-derived EVs analysis have gained increasing interest from multidisciplinary researchers [264]. In the field of vision science, two studies have been identified that employ single-vesicle analysis, in which both EVs were isolated from aqueous humor. One study revealed CD63^+^ EVs as a potential biomarker for pediatric retinoblastoma [187]. Another showed an increased proportion of CD63/81⁺ and CD9/63/81⁺ EVs in uveal melanoma patients following brachytherapy [144]. Compared to bulk analysis of EVs, the emerging single-vesicle analysis provides key advantages. Specifically, it enables the direct characterization of EVs heterogeneity, protein profiling with minimal sample volumes (< 20 μL), enhances drug delivery efficacy, and uncovers distinct molecular states regulating EVs function and transport [264, 265]. Under such circumstances, the application of single-vesicle imaging and analysis in vision science is anticipated to expand. 

### Potential promising EVs source: organoids and plants

Nowadays, the retinal organoid is the only ocular organoid explored for its derived EVs in visual science. Given that organoids closely mimic the structure and function of natural human organs, they serve as an effective model for in vivo organ development and disease [266]. In this regard, it is estimated that future research will increasingly focus on EVs derived from other ocular organoids, such as corneal organoids [267, 268], corneal limbal organoids [269], corneal epithelial organoids [270], and human retinal stem cells [271]. Moreover, studies utilizing EVs from retinal organoids are also expected to expand. 

Despite extensive research on various EVs sources, plant-EVs remain unexplored in vision science. Plant EVs, biogenic and morphologically similar to mammalian EVs, offer distinctive advantages: they are less immunogenic than humans, highly stable under diverse environmental conditions, and resistant to enzymatic degradation due to their unique physicochemical properties [272, 273]. Notably, edible plants are naturally abundant, biocompatible, and biodegradable. Therefore, EVs from plants, particularly those from edible plants, may present a promising cell-free therapeutic option for ocular disorders.

## Limitations

First, data in this study were collected from four globally recognized databases, which may have resulted in the exclusion of relevant data from other sources. Nonetheless, such data sources are often difficult to access publicly or lack standardized subfields and metadata that are available in Web of Science, Scopus, Embase, and PubMed. Second, while we integrated VOSviewer´s visualization capabilities with CiteSpace´s analytical depth for a holistic study view of EVs original research in visual science, it may lead to overreliance without validation. Utilizing complementary tools such as Bibliometrix (R package) could strengthen future analyses. Third, the overall extent of the retinal degeneration cluster was reduced as the studies of diabetic retinopathy and AMD were assigned to their own cluster, even though they are two of the four types of retinal degeneration. Fourth, studies on microvesicles and exosomes are often collectively labeled as EVs, either for analytical simplicity or within discussions, making it difficult to distinguish between them.

Moreover, the focus on thematic clusters may have led to the omission of certain high-citation topics, such as glaucoma and uveitis (Suppl. Fig. 2). However, this underrepresentation is not due to the result of the clustering algorithms or inclusion thresholds, as both topics remained marginal even after parameter adjustments. Notably, the knowledge base specific to glaucoma or uveitis has not yet emerged (Fig. 6), which may explain their absence from thematic clusters within the research frontiers. The relatively high frequency of citations of these topics may, however, be attributed to other factors. For example, glaucoma, which is closely linked to IOP and RGCs damage, is currently dispersed across clusters of TM, aqueous humor, and retinal degeneration. Similarly, uveitis-related studies are embedded within clusters on aqueous humor and bioengineered EVs-based therapeutic applications. These patterns suggest that both disease areas represent developing but not yet consolidated research trajectories.

## Conclusion

Significant progress has been made in understanding and applying EVs in vision science since 2003, culminating in the establishment of a stable and mature knowledge base by 2023. While most ocular thematic clusters are now well-established, the potential roles of EVs in conditions such as keratitis, high myopia–related retinal lesions, retinitis pigmentosa, retinoblastoma, and thyroid eye disease remain underrepresented and warrant further investigation. Notably, EVs-based clinical therapeutic outcomes remain vastly lacking. However, advances in single-vesicle analysis, EVs bioengineering, EVs purification, organ-on-chip systems, artificial intelligence-driven prediction of EVs bioactivity, as well as the use of EVs derived from three-dimensional cultures, ocular organoids, plants, or EV-track platform may boost the EVs clinical translation in vision science.

## Supplementary Information

Additional file1

## Data Availability

No datasets were generated or analysed during the current study.

## Abbreviations

AAV: Adeno-associated virus
AMD: Age-related macular degeneration
AH: Aqueous humor
CNV: Choroidal neovascularization
CEndCs: Corneal endothelial cells
CEpiCs: Corneal epithelial cells
CSSCs: Corneal stromal stem cells
DED: Dry eye disease
DM: Diabetic mellitus
DR: Diabetic retinopathy
EVs: Extracellular vesicles
EAU: Experimental autoimmune uveitis
ECM: Extracellular matrix
EMT: Epithelial-mesenchymal transition
iPSCs: Human induced pluripotent stem cells
LECs: Lens epithelial cells
lnc RNA: Long non-coding RNA
MMPs: Matrix metalloproteinases
MS: Mass spectrometry
MSCs: Mesenchymal stem cells
NGM: Next-generation sequencing
NPEC: Non-pigmented epithelial cell
NTA: Nanoparticle tracking analysis
OIR: Oxygen-induced retinopathy
PCO: Posterior capsular opacification
POAG: Primary open-angle glaucoma
PVR: Proliferative vitreoretinopathy
PTEN: Phosphatase and tensin homolog
RD: Retinal degeneration
RGCs: Retinal ganglion cells
RNA-seq: RNA-sequencing
ROS: Reactive oxygen species
RPE: Retinal pigment epithelium
TM: Trabecular meshwork
UM: Uveal melanoma
VEGF: Vascular endothelial growth factor
VH: Vitreous humor
