# Supplementary material for: Scientometric analysis of extracellular vesicles in vision science (up to 2024)
Source: J Nanobiotechnology. 2025 Oct 10;23:654. doi: 10.1186/s12951-025-03703-5 (PMC12514847; doi:10.1186/s12951-025-03703-5)
Supplement: Supplementary file 1 — Additional file1 [file 12951_2025_3703_MOESM1_ESM.docx]

**Supplementary Methods section**

**1 Database-Specific Query Syntax**

**1.1 Web of Science Core Collection:**

Search in: Web of Science Core Collection

Editions: Science Citation Index Expanded (SCI-EXPANDED)

(TI=(exosome* OR microvesicle* OR "extracellular vesicle*") OR AB=(exosome* OR microvesicle* OR "extracellular vesicle*") OR AK=(exosome* OR microvesicle* OR "extracellular vesicle*")) AND (TI=(eye OR eyebrow* OR eyelid* OR eyelash* OR "anterior eye segment" OR "anterior chamber" OR "aqueous humor" OR "ciliary body" OR conjunctiva OR cornea OR iris OR lens OR "meibomian glands" OR "lacrimal apparatus" OR "nasolacrimal duct" OR "pigment epithelium" OR "posterior eye segment" OR "vitreous humor" OR retina OR "blood-retinal barrier" OR "macula lutea" OR "optic disk" OR "retinal neurons" OR sclera OR "tenon capsule" OR uvea OR "blood-aqueous barrier" OR choroid OR "optic nerve") OR AB=(eye OR eyebrow* OR eyelid* OR eyelash* OR "anterior eye segment" OR "anterior chamber" OR "aqueous humor" OR "ciliary body" OR conjunctiva OR cornea OR iris OR lens OR "meibomian glands" OR "lacrimal apparatus" OR "nasolacrimal duct" OR "pigment epithelium" OR "posterior eye segment" OR "vitreous humor" OR retina OR "blood-retinal barrier" OR "macula lutea" OR "optic disk" OR "retinal neurons" OR sclera OR "tenon capsule" OR uvea OR "blood-aqueous barrier" OR choroid OR "optic nerve") OR AK=(eye OR eyebrow* OR eyelid* OR eyelash* OR "anterior eye segment" OR "anterior chamber" OR "aqueous humor" OR "ciliary body" OR conjunctiva OR cornea OR iris OR lens OR "meibomian glands" OR "lacrimal apparatus" OR "nasolacrimal duct" OR "pigment epithelium" OR "posterior eye segment" OR "vitreous humor" OR retina OR "blood-retinal barrier" OR "macula lutea" OR "optic disk" OR "retinal neurons" OR sclera OR "tenon capsule" OR uvea OR "blood-aqueous barrier" OR choroid OR "optic nerve")). Timespan: 1990-01-01 to 2024-12-31 (Publication Date)

**1.2 Embase (searched in Title/Abstract/Keywords):**

(exosome*:ti,ab,kw OR microvesicle*:ti,ab,kw OR 'extracellular vesicle*':ti,ab,kw) AND (eye:ti,ab,kw OR eyebrow*:ti,ab,kw OR eyelid*:ti,ab,kw OR eyelash*:ti,ab,kw OR 'anterior eye segment':ti,ab,kw OR 'anterior chamber':ti,ab,kw OR 'aqueous humor':ti,ab,kw OR 'ciliary body':ti,ab,kw OR conjunctiva:ti,ab,kw OR cornea:ti,ab,kw OR iris:ti,ab,kw OR lens:ti,ab,kw OR 'meibomian glands':ti,ab,kw OR 'lacrimal apparatus':ti,ab,kw OR 'nasolacrimal duct':ti,ab,kw OR 'pigment epithelium':ti,ab,kw OR 'posterior eye segment':ti,ab,kw OR 'vitreous humor':ti,ab,kw OR retina:ti,ab,kw OR 'blood-retinal barrier':ti,ab,kw OR 'macula lutea':ti,ab,kw OR 'optic disk':ti,ab,kw OR 'retinal neurons':ti,ab,kw OR sclera:ti,ab,kw OR 'tenon capsule':ti,ab,kw OR uvea:ti,ab,kw OR 'blood-aqueous barrier':ti,ab,kw OR choroid:ti,ab,kw OR 'optic nerve':ti,ab,kw) AND [1967-2024]/py

**1.3 Scopus (searched in Title, Abstract, Keywords):**

TITLE-ABS-KEY(exosome* OR microvesicle* OR "extracellular vesicle*") AND TITLE-ABS-KEY(eye OR eyebrow* OR eyelid* OR eyelash* OR "anterior eye segment" OR "anterior chamber" OR "aqueous humor" OR "ciliary body" OR conjunctiva OR cornea OR iris OR lens OR "meibomian glands" OR "lacrimal apparatus" OR "nasolacrimal duct" OR "pigment epithelium" OR "posterior eye segment" OR "vitreous humor" OR retina OR "blood-retinal barrier" OR "macula lutea" OR "optic disk" OR "retinal neurons" OR sclera OR "tenon capsule" OR uvea OR "blood-aqueous barrier" OR choroid OR "optic nerve")) AND PUBYEAR BEF 2025

**1.4 PubMed (searched in Title/Abstract):**

((exosome*[Title/Abstract] OR microvesicle*[Title/Abstract] OR "extracellular vesicle*"[Title/Abstract]) AND (eye[Title/Abstract] OR eyebrow*[Title/Abstract] OR eyelid*[Title/Abstract] OR eyelash*[Title/Abstract] OR "anterior eye segment"[Title/Abstract] OR "anterior chamber"[Title/Abstract] OR "aqueous humor"[Title/Abstract] OR "ciliary body"[Title/Abstract] OR conjunctiva[Title/Abstract] OR cornea[Title/Abstract] OR iris[Title/Abstract] OR lens[Title/Abstract] OR "meibomian glands"[Title/Abstract] OR "lacrimal apparatus"[Title/Abstract] OR "nasolacrimal duct"[Title/Abstract] OR "pigment epithelium"[Title/Abstract] OR "posterior eye segment"[Title/Abstract] OR "vitreous humor"[Title/Abstract] OR retina[Title/Abstract] OR "blood-retinal barrier"[Title/Abstract] OR "macula lutea"[Title/Abstract] OR "optic disk"[Title/Abstract] OR "retinal neurons"[Title/Abstract] OR sclera[Title/Abstract] OR "tenon capsule"[Title/Abstract] OR uvea[Title/Abstract] OR "blood-aqueous barrier"[Title/Abstract] OR choroid[Title/Abstract] OR "optic nerve"[Title/Abstract])) AND (("1996/01/01"[Date - Publication] : "2024/12/31"[Date - Publication]))

**2. Data Normalization and Standardization**

**Table S1-Data normalization and standardization of country**

| **label** | **replace by** | **label** | **replace by** |
| --- | --- | --- | --- |
| Hong Kong | China | Taiwan | China |
| Republic of Korea | South Korea | U Arab Emirates | United Arab Emirates |
| Korea | South Korea | United Kingdom | England |
| Scotland | England | United States | USA |
| Turky | Turkey | Wales | England |
| Turkiye | Turkey |  |  |

**Table S2- Data normalization and standardization of institutions**

| **label** | **replace by** | **label** | **replace by** |
| --- | --- | --- | --- |
| a*star - institute of med biology | A*STAR | Instituto de Salud Carlos III | ISCIII |
| a*star - institute of molecular and cell biology | A*STAR | Manipal Academy of Higher Education | MAHE |
| agency for sci tech and research | A*STAR | National Institutes of Health | NIH |
| centro of IBRBBN | CIBER | National Eye Institute | NEI |
| ciberbbn | CIBER | Penn State Hershey College of Medicine | PCSHE |
| consejo superior de investigaciones cientificas | CSIC | People's Liberation Army General Hospital | PLAGH |
| csic - instituto of biomedicina of valencia | CSIC | Spanish National Cancer Research Center | CNIO |

IBRB：Investigación Biomédica en Red of Bioingeniería, Biomateriales y Nanomedicina

**Table S3- Data normalization and standardization of author names**

| **label** | **replace by** | | **label** | **replace by** |
| --- | --- | --- | --- | --- |
| belfort, rubens | | belfort, rubens n. | mei, heng-jun | mei, hengjun |
| burnier, julia valdemarin | | burnier, julia v. | mishra, dilip kumar | mishra, dilip k. |
| cai, jinyu | | cai jinyu | park, susanna | park, susanna s |
| calero, c | | calero-medina, c | perkumas, k. m | perkumas, kristin m. |
| medina, cc | | calero-medina, c | rohrer, barbel | rohrer, bärbel |
| medina, cmc | | calero-medina, c | skiba, nikolai | skiba, nikolai p. |
| canto-soler, m. valeria | | canto-soler, maria v. | stamer, w daniel | stamer, w. d. |
| ciolino, joseph | | ciolino, joseph b. | torres, leianne | torres, leianne a. |
| cortinas, john | | cortinas, john a. | wroblewska, joanna patrycja | wróblewska, joanna p. |
| dos santos, aurelie | | dos santos, aurélie | xiaomin zhang | zhang xiaomin |
| funderburgh, martha | | funderburgh, martha l. | zieske, james | zieske, james d. |
| handa, j, t | | handa, james t. |  |  |

**Table S4- Data normalization and standardization of journal sources**

| **label** | **replace by** | **label** | **replace by** |  |
| --- | --- | --- | --- | --- |
| biochimica et biophysica acta | biochim biophys acta | j cell mol med | JCMM | |
| cell metabol | cell metab | j cell biol | journal of cellular biochemistry | |
| curr eye res | current eye research | j biol chem | journal of biological chemistry | |
| exp cell res | experimental cell research | j clin invest | j. clin. investig | |
| exp eye res | experimental eye research | prog retin eye res | progress in retinal and eye research | |
| int j mol sci | IJMS | sci rep | scientific reports | |
| invest ophthalmol vis sci | IOVS | stem cell rep | stem cell reports | |
| investig ophthalmol vis sci | IOVS | stem cell res | stem cell research & therapy | |
| proc natl acad sci usa | PNAS | stem cell res ther | stem cell research & therapy | |
| proc. natl. acad. sci. u. s. a. | PNAS | stem cell. | stem cells | |
| j extracell vesicles | JEV | stem cells int | stem cells international | |
| j control release | JCR | stem cells transl med | stem cells translational medicine | |
| j control rel | JCR | transl vis sci technol | TVST | |

IJMS: international journal of molecular sciences. IOVS: investigative ophthalmology & visual science. JCMM: journal of cellular and molecular medicine. JEV: journal of extracellular vesicles. JCR: journal of controlled release. PNAS: proceedings of the national academy of sciences of the united states of america. TVST: translational vision science & technology.

**Table S5- Data normalization and standardization for author keywords**

| **label** | **replace by** | **label** | **replace by** | |
| --- | --- | --- | --- | --- |
| *extracellular vesicles | EVs | mesenchymal stromal cells | MSCs |  |
| *micrornas | miRNA | mesenchymal stromal cells (mscs) | MSCs |  |
| adipose mscs | MSCs | messenger rna | mRNA |  |
| adipose-derived mesenchymal stem cell | MSCs | messenger-rna | mRNA |  |
| adipose-derived stem cells | MSCs | messenger-rnas | mRNA |  |
| adipose-tissue | MSCs | micro rnas | miRNA |  |
| age related macular degeneration | AMD | microrna | miRNA |  |
| age-related macular degeneration | AMD | microrna expression | miRNA |  |
| age-related macular degeneration (amd) | AMD | micrornas | miRNA |  |
| amd | AMD | micro-rnas | miRNA |  |
| animals | animal | microscopy electron transmission | TEM |  |
| aqueous humor (ah) | aqueous humor | microvesicle | EVs |  |
| aqueous-humor | aqueous humor | microvesicles | EVs |  |
| arpe-19 cells | arpe-19 | milk exosome | EVs |  |
| bdnf-trkb pathway | BDNF/TrkB | milk-derived exosomes | EVs |  |
| biological marker | biomarker | mirnas | miRNA |  |
| biomarkers | biomarker | mitochondrial membrane potential | MtMP |  |
| blotting, western | WB | mitochondrion | mitochondria |  |
| bone marrow | MSCs | mitogen activated protein kinase | MAPK |  |
| bone marrow mesenchymal stem cell | MSCs | mmp14 | MMP-14 |  |
| bone marrow mesenchymal stem cells | MSCs | monocyte chemotactic protein 1 | MCP-1 |  |
| bone marrow-derived mesenchymal stem cell | MSCs | mouse | mice |  |
| bone marrow-derived stem cells | MSCs | mrna expression level | mRNA |  |
| bone-marrow | MSCs | msc-evs | EVs |  |
| cataracts | cataract | msc-sev | EVs |  |
| cataract-surgery | cataract | nanoparticle tracking analysis | NTA |  |
| cell line tumor | tumor cell line | next-generation sequencing | NGS |  |
| cell proliferation assay | cell proliferation | next generation sequencing | NGS |  |
| cell-cell communication | cellular crosstalk | nf-kappa b signaling pathway | NF-κβ |  |
| cell-derived exosomes | EVs | nlrp3 inflammasome | NLRP3 |  |
| cell-derived extracellular vesicles | EVs | non-pigmented ciliary epithelium | NPEC |  |
| cell-free immunomodulatory therapy | cell-free therapy | non pigmented ciliary epithelium | NPEC |  |
| cells cultured | cell culture | optical coherence tomography | OCT |  |
| cellular communication | cellular crosstalk | oxygen-induced retinopathy | OIR |  |
| cgas-sting pathway | cGAS_STING | oxygen induced retinopathy | OIR |  |
| circular rna | circRNA | photon correlation spectroscopy | PCS |  |
| coculture techniques | coculture | photoreceptor | photoreceptors |  |
| complementary dna | cDNA | photoreceptor cells | photoreceptors |  |
| cornea epithelium | corneal epithelial cells | pi3k/akt pathway | pi3k/akt |  |
| corneal endothelium | corneal endothelial cells | pi3k/akt/foxo3 pathway | pi3k/akt |  |
| corneal epithelial cell | corneal epithelial cells | pi3k/akt/mtor pathway | pi3k/akt |  |
| corneal injury (ci) | corneal injury | pi3k-akt signaling pathway | pi3k/akt |  |
| corneal stroma | corneal stem cells | pigment epithelial-cells | RPE |  |
| corneal stromal cells | corneal stem cells | pigment epithelium | RPE |  |
| corneal stromal stem cells | corneal stem cells | pigment epithelium-derived factor | PEDF |  |
| cytokine | cytokines | plasma sevs | EVs |  |
| dendritic cell | dendritic cells | plasma-derived sevs | EVs |  |
| diabetes | DM | platelet extracellular vesicles | EVs |  |
| diabetes mellitus | DM | posterior capsule opacification | PCO |  |
| diabetic-retinopathy | diabetic retinopathy | primary-open angle glaucoma | POAG |  |
| disease model | disease models | protein | proteins |  |
| drug-delivery | drug delivery | protein expression | proteins |  |
| drug-delivery vehicles | drug delivery | protein expression level | proteins |  |
| dry eye disease | dry eye | proteomic analysis | proteomic |  |
| dry eye syndrome | dry eye | proteomic profiles | proteomic |  |
| endothelial cells | endothelium cells | proteomics | proteomic |  |
| endothelial-cells | endothelium cells | proliferative vitreoretinopathy | PVR |  |
| endothelium cell | endothelium cells | rat | rats |  |
| enzyme linked immunosorbent assay | ELISA | rat model | rats |  |
| enzyme-linked immunosorbent assay | ELISA | reactive oxygen species | ROS |  |
| epithelial-mesenchymal transition | EMT | real time polymerase chain reaction | RT-PCR |  |
| epithelial mesenchymal transition | EMT | real-time polymerase chain reaction | RT-PCR |  |
| epithelial-mesenchymal transformation | EMT | retina degeneration | retinal degeneration |  |
| ev | EVs | retina ganglion cell | RGCs |  |
| exosomal mirna | miRNA | retina ganglion cells | RGCs |  |
| exosome | EVs | retinal degenerations | retinal degeneration |  |
| exosomes | EVs | retinal degenerative diseases | retinal degeneration |  |
| exososomes | EVs | retinal ganglion cell | RGCs |  |
| experimental autoimmune uveitis (eau) | EAU | retinal ganglion cells | RGCs |  |
| experimental autoimmune uveitis | EAU | retinal ganglion-cells | RGCs |  |
| extracellular exosome | EVs | retinal ischemia reperfusion | retinal ischemia |  |
| extracellular vesicle | EVs | retinal muller cells | Müller cells |  |
| extracellular vesicle (ev) | EVs | retinal nerve fiber layer thickness | RNFL thickness |  |
| extracellular vesicles | EVs | retinal pigment epithelia | RPE |  |
| extracellular vesicles (evs) | EVs | retinal pigment epithelial cell | RPE |  |
| extracellular vesicles (exos) | EVs | retinal pigment epithelial cell (rpe) | RPE |  |
| extracellular vesicles secretion | EVs | retinal pigment epithelial cells | RPE |  |
| extracellular-matrix | ECM | retinal pigment epithelium | RPE |  |
| extracellular matrix | ECM | retinal pigment epithelium cells | RPE |  |
| fibroblast | fibroblasts | retinal-pigment epithelium | RPE |  |
| gamma interferon | IFN-γ | retinitis-pigmentosa | retinitis pigmentosa |  |
| ganglion-cells | RGCs | rna | RNA |  |
| gingival mesenchymal stem cells | MSCs | rna sequencing | RNA-Seq |  |
| glaucoma diagnosis | glaucoma | rna messenger | RNA |  |
| hek293t cell line | HEK293T cells | rnas | RNA |  |
| human amniotic mesenchymal stem cells | MSCs | rpe cells | RPE |  |
| human umbilical cord mesenchymal stem cell | MSCs | small extracellular vesicle | EVs |  |
| human umbilical cord mscs | MSCs | small extracellular vesicles | EVs |  |
| human umbilical-cord | MSCs | small interfering rna | siRNA |  |
| il-1 beta | iL-1β | stem cell | stem cells |  |
| immunofluorescence | IF | stem cell-derived ev | EVs |  |
| immunohistochemistry | IHC | stem-cells | stem cells |  |
| immunomodulatory | immunomodulation | stroma cell | stromal cells |  |
| immunoprecipitation | IP | stroma cells | stromal cells |  |
| induced pluripotent stem cells | iPSCs | tandem mass spectrometry | Tandem MS |  |
| inhibitors | inhibitor | t-cell | t cells |  |
| interleukin 1beta | iL-1β | tear | tears |  |
| interleukin 6 | iL-6 | therapeutics | therapy |  |
| interleukin 8 | iL-8 | thy 1 membrane glycoprotein | Thy-1 |  |
| intraocular-pressure | intraocular pressure | transmission electron microscopy | TEM |  |
| keratocyte | keratocytes | tumor necrosis factor | TNF |  |
| lens epithelial cell | lens epithelial cells | umbilical cord | MSCs |  |
| long non-coding rna | lnc RNA | umbilical vein endothelial cell | HUVECs |  |
| long non-coding rnas | lnc RNA | uvea melanoma | uveal melanoma |  |
| mass spectrometry analysis | mass spectrometry | vascular endothelial growth factor | VEGF |  |
| mass-spectrometry | mass spectrometry | vascular endothelial growth factor a | VEGF α |  |
| mechanism | mechanisms | vesicles | EVs |  |
| mesenchymal cells | MSCs | vitreous | vitreous humor |  |
| mesenchymal stem cell | MSCs | vitreous humor (vh) | vitreous humor |  |
| mesenchymal stem cells | MSCs | vogt-koyanagi-harada disease | VKH |  |
| mesenchymal stem cells (mscs) | MSCs | vogt-koyanagi-harada | VKH |  |
| mesenchymal stem/stromal cells | MSCs | vogt-koyanagi-harada syndrome | VKH |  |
| mesenchymal stem-cells | MSCs | western blotting | WB |  |

**Suppl. Table 6- Clinical trials of EVs in vision science from the Cochrane Library**

| **Ocular conditions** | **EVs source** | **Year** | **Country** | **Registration ID** |
| --- | --- | --- | --- | --- |
| Dry eye | hUMSCs | 2020 | China | ChiCTR2000031188 |
| Retinitis pigmentosa | hUMSCs | 2022 | Turkey | ClinicalTrials.gov ID NCT05413148 |
| Dry eye | WJ-MSCs | 2022 | Iran | IRCT20211102052948N1 |
| Dry eye | limbal-derived MSCs | 2023 | Iran | IRCT20231022059814N1 |
| Corneal epithelial defects | WJ-MSCs | 2024 | Iran | IRCT20240706062337N1 |

hUMSCs: umbilical cord MSCs. WJ-MSCs: Wharton jelly-derived MSCs. Data accessed on 23 April 2025.

**Suppl. Table-7 Limitations, gaps & future opportunities of EVs in ocular disease**

| **Research area** | **Current limitations** | **Gap & Future Opportunities** |
| --- | --- | --- |
| **Angiogenesis** | Scarcity of EVs involved pathophysiology.  Limited EVs cargo signaling mechanism. | EVs VEGF-regulatory biomarkers.  Engineering EVs loaded with anti-angiogenic cargoes. |
| **Cornea injury**  **Dry eye disease** | Lack/paucity of biomarkers.  Unclear EVs biodistribution and retention.  Lack EVs-associated pathophysiology. | EVs-associated pathophysiology.  Engineering EVs to selectively target corneal layer.  Engineering EVs with stable ocular retention |
| **IOP / glaucoma / RGCs-damage** | Unclear impact of IOP levels on EVs properties.  Scarcity of EVs-mediated glaucomatosa RD. | Engineering EVs to selectively target trabecular meshwork / SCE cells / RGCs |
| **Diabetic retinopathway** | Biomarker shortage for therapy monitoring.  Potential EVs role in disease exacerbation. | EVs cargo variation in early / late disease stages  Engineering EVs with sustained retinal delivery |
| **AMD** | Scarcity of EVs in wet AMD pathophysiology. EVs-mediated RPE-photoreceptors crosstalk. | EVs-mediated RPE–choroidal vascular crosstalk.  Engineering EVs with sustained retinal delivery. |
| **Uveal melanoma** | No pre-metastatic EVs pathophysiology.  Scarcity of early detection biomarkers. | EVs cargo variation pre-/post disease metastasis.  EVs role in resistance treatment response. |
| **Endophthalmitis** | Limited EVs cargo signaling mechanism.  Limited pathogen scope. | EVs diagnostic and therapeutic potential biomarkers.  Engineering EVs for refractory endophthalmitis. |
| **RD** | Paucity of EVs-involved pathophysiology.  Biomarker shortage for early detection. | EVs cargo changes in early / late disease stages.  Engineering EVs to selectively target retinal cells. |

AMD: age-related macular degeneration. IOP: intraocular pressure. RD: retinal degeneration. RGCs: retinal ganglion cells. RPE: retinal pigment epithelial. SCE cells: Schlemms canal endothelial cells. VEGF: vascular endothelial growth factor.


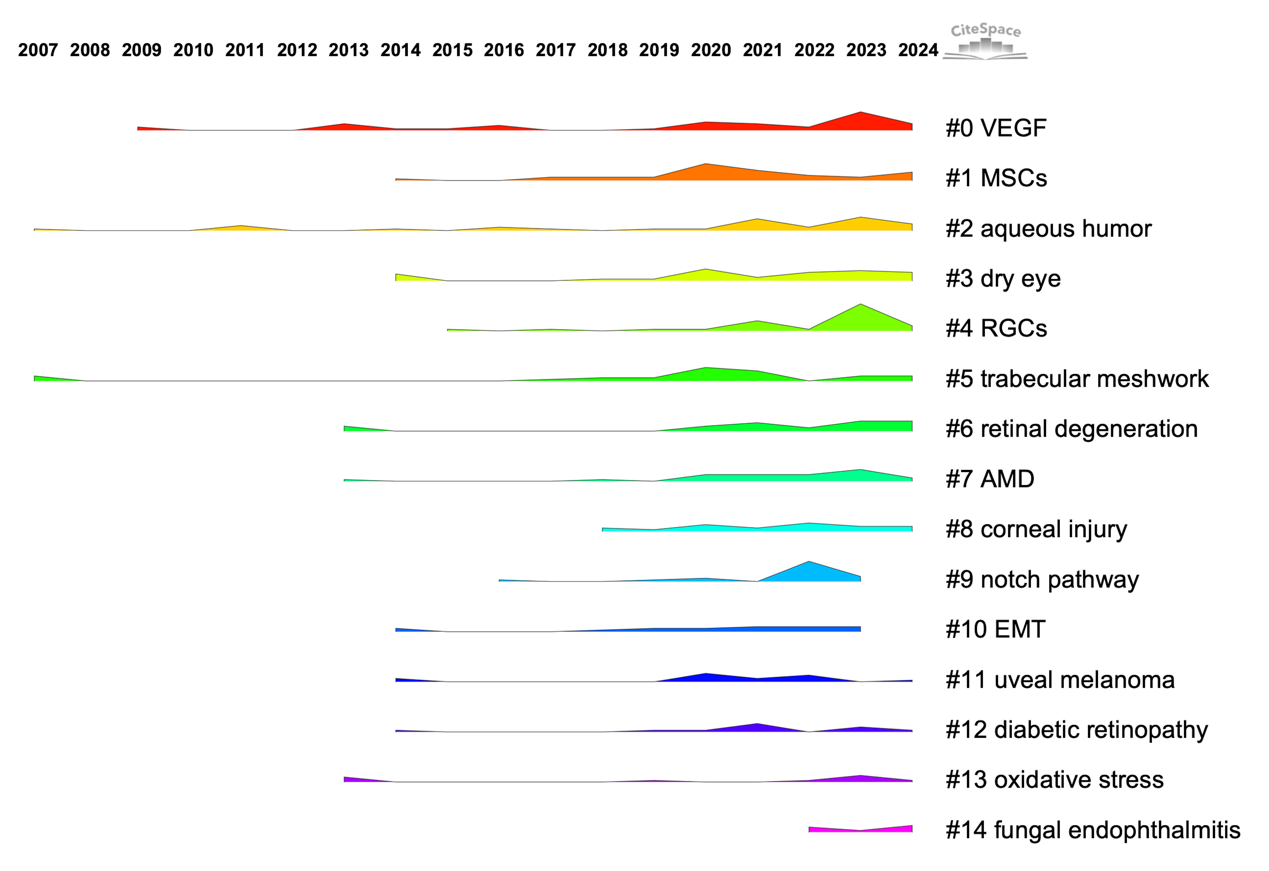


**Suppl. Figure 1.-** The thematic landscape of research frontiers on EVs in vision science.


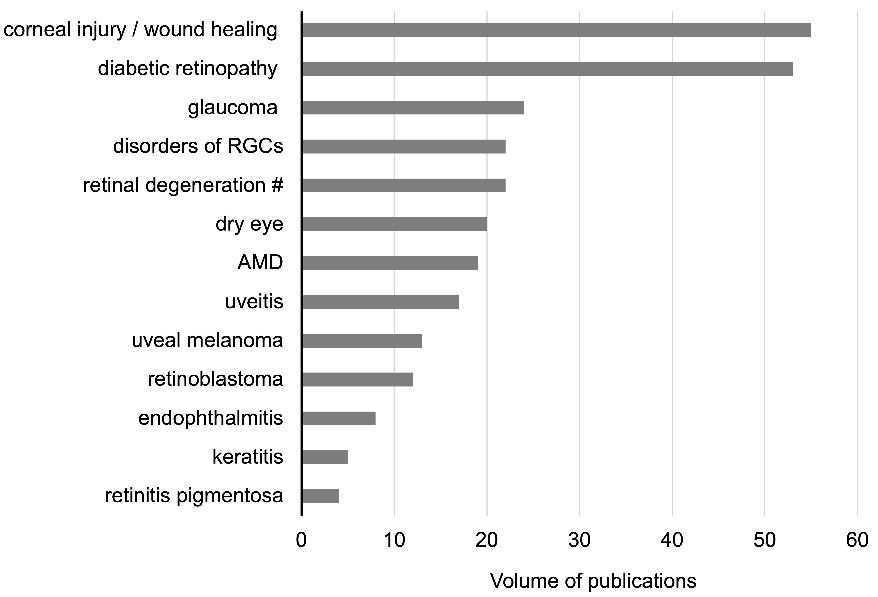


**Suppl. Figure 2.-** Distribution of publications on EVs in ocular disease research. AMD: age-related macular degeneration. RGCs: retinal ganglion cells. #: The study did not specify the particular type of retinal degeneration examined.
